# Acupuncture for post-stroke depression: a systematic review and network meta-analysis

**DOI:** 10.1186/s12888-023-04749-1

**Published:** 2023-05-04

**Authors:** Wai Lam Ching, Hui Juan Li, Jianwen Guo, Liang Yao, Janita Chau, Suzanne Lo, Chun Sum Yuen, Bacon Fung Leung Ng, Edwin Chau-Leung Yu, Zhaoxiang Bian, Alexander Y Lau, Linda LD Zhong

**Affiliations:** 1 Biomedical Sciences and Chinese Medicine, School of Biological Sciences, Nanyang Technological University, Singapore, 637551 Singapore; 2 Hong Kong Chinese Medicine Clinical Study Centre, School of Chinese Medicine, Hong Kong Baptist University, Hong Kong, Hong Kong SAR China; 3 Brain Center, Guangdong Provincial Hospital of Traditional Chinese Medicine, Guangdong University of Chinese Medicine, Guangzhou, China; 4 Department of Health Research Methods, Evidence and Impact, McMaster University, Hamilton, ON Canada; 5 The Nethersole School of Nursing, Faculty of Medicine, the Chinese University of Hong Kong, Ma Liu Shui, Hong Kong SAR China; 6 School of Chinese medicine, Hong Kong Baptist University, Kowloon, Hong Kong SAR China; 7 Department of Rehabilitation Sciences, Hong Kong Polytechnic University, Kowloon, Hong Kong SAR China; 8 Hong Kong Association for Integration of Chinese-Western Medicine Limited, Kowloon, Hong Kong SAR China; 9 Department of Medicine and Therapeutics and Hong Kong Institute of Integrative Medicine, Prince of Wales Hospital, Faculty of Medicine, the Chinese University of Hong Kong, Kowloon, Hong Kong SAR China

**Keywords:** Acupuncture, Post-stroke depression, Network meta-analysis

## Abstract

**Background:**

Patients with post-stroke depression (PSD) usually experience anxiety, hopelessness, and insomnia, which have a negative impact on their daily activities and post-stroke rehabilitation. Acupuncture (AC), as a minimally invasive technique, has become a popular choice for improving depression symptoms. However, it is still unclear which therapy is associated with the best outcomes for PSD. In this review, we aimed to explore the impact of AC in alleviating symptoms of PSD and to evaluate the difference in effectiveness between AC combined with pharmacotherapies and various non-pharmacotherapies.

**Methods:**

Six databases and three clinical trials registration platforms were searched from inception to March 2023. Randomized clinical trial comparing needle-based AC with pharmacotherapy, and other non-pharmacotherapy or invalid group were included. Two independent reviewers identified eligible studies, and collected data using a pre-made form. A Bayesian network meta-analysis was conducted to assess and compare different techniques using RStudio 3.6.0 with the package ‘GEMTC’ V.0.8.1. The primary outcome was the efficacy for PSD assessed by scales measuring depressive symptoms. The secondary outcomes were effectiveness for neurological function and the quality of life. The ranking probabilities for all treatment interventions was performed using the Surface Under the Cumulative Ranking curve (SUCRA). The risk of bias was assessed by using the Revised Cochrane Risk of Bias tool 2.

**Results:**

Sixty-two studies, involving 5308 participants published from 2003 to 2022, were included. The results showed that compared with western medicine (WM) (defined as pharmacotherapy for PSD), AC alone or with repetitive transcranial magnetic stimulation (RTMS), Traditional Chinese medicine (TCM) alone or with WM, were superior for alleviating depression symptoms. Compared to Usual Care, AC alone or plus other therapies could significantly decrease scores on the Hamilton Depression Rating scale. According to result of SUCRA, AC plus RTMS had the highest probability of improving depressive symptoms with a probability of 49.43%.

**Conclusions:**

The results of this study indicate that AC alone or combined with other therapies appears to be effective in improving depression symptoms of stroke survivors. Moreover, in comparison to WM, AC alone or plus RTMS, TCM, TCM with WM, or WM, were more effective in improving depression symptoms of PSD. Also, AC with RTMS seems to be the most effective with the highest probability.

**Registration:**

This study was registered in the International Prospective Register of Systematic Reviews (PROSPERO) database in November 2020 and updated in July 2021. The registration number is CRD42020218752.

**Supplementary Information:**

The online version contains supplementary material available at 10.1186/s12888-023-04749-1.

## Background

Stroke is the second leading cause of death, according to the World Health Organization (WHO) in 2019, responsible for approximately 11% of total deaths [1]. Moreover, it is also one of the leading causes of disability worldwide, and causes the loss of a great number of healthy life-years due to serious post-stroke sequelae (such as paralysis, aphasia, dysphagia, epilepsy, and cognitive difficulty) [2–5]. Facing severe symptoms and different physical disabilities, stroke survivors might face tremendous health challenges, and they are more likely to suffer from psychological disorders. Depression is one of the most common complications after stroke, with a prevalence of 30–33% [6–9]. Patients with post-stroke depression (PSD) usually experience anxiety, hopelessness, unwillingness to communicate, and insomnia, which could have a negative impact on daily activities and post-stroke rehabilitation [10]. Moreover, it is suggested that PSD is associated with an increased risk of mortality in stroke survivors [6]. Therefore, it is quite crucial to devise an effective and safe treatment for PSD. Selective serotonin reuptake inhibitors (SSRIs) are commonly recommended as the first-line pharmacological treatments [11]. However, there still exists a debate on whether SSRIs could increase the risk of intracerebral hemorrhage and subsequent stroke [12, 13].

Acupuncture (AC), as a minimally invasive technique, has been widely used for improving symptoms of a variety of health problems in China and worldwide [14–16]. Many published studies have investigated the effectiveness of AC for PSD. One meta-analysis by Xin Yan Zhang etc. included seven studies comparing the effectiveness rate of AC with control group in alleviating the symptoms of PSD. Their results supported that AC was an effective and safe treatment for PSD[17]. (RR 1.16, 95% CI 1.08–1.24). Another study by Xue Wang, etc. evaluated the effectiveness of AC combined with western medicine for PSD based on 24 studies. The meta-analysis showed AC combined with fluoxetine was superior to fluoxetine alone for relieving depressive symptoms [18]. Moreover, more clinical trials evaluated whether there was a better effect when AC combined with other non-pharmacotherapies for PSD. The study by Zhang LIN etc. investigated the efficacy of AC plus Tai Chi in recovering the neurological function and treating depression in PSD [19]. Another study compared the difference between AC combined plus cognitive therapy with paroxetine [20]. The authors of Yaqun WANG etc. enrolled 103 participants to evaluate the effect of AC plus Jieyu Qingxin Decotion (a Traditional Chinese herbal medicine) combined compared with paroxetine.

In this review, we aimed to explore the true effect of AC in alleviating symptoms of PSD based on both direct and indirect evidence using the network meta-analysis (NMA) method [21]. Moreover, we also aimed to evaluate the difference in effectiveness between AC with pharmacotherapies and other non-pharmacotherapies in order to provide optimized guidance and advice for clinical personnel.

## Methods

This review was reported in accordance with the Preferred Reporting Items for Systematic Review and Meta-Analyses (PRISMA) for Network Meta-Analyses (Appendix 1), [22] and was registered in the International Prospective Register of Systematic Reviews (PROSPERO) database in November 2020 and updated in July 2021 (Registration no.: CRD42020218752) [23].

### Literature search

The Cochrane Library, PubMed, EMBASE, China National Knowledge Infrastructure (CNKI), Wanfang Database, Chongqing VIP Database (CQVIP) were searched for systematic reviews of published articles from inception to March 2023. The clinical trials registration platform and websites were additionally searched, including the International Clinical Trials Registry Platform (ICTRP) (https://www.who.int/clinical-trials-registry-platform), ClinicalTrials.gov (https://www.clinicaltrials.gov/), ISRCTN registry (http://www.isrctn.com/editAdvancedSearch), https://scholar.google.com.tw/, https://xueshu.baidu.com/, https://www.geenmedical.com/. There was no restriction in language or publication year. Besides, the references of relevant reviews and systematic reviews were retrieved to search for potential eligible studies. The search strategy of this study is shown in Supplementary Appendix 2.

### Inclusion Criteria and Exclusion Criteria

Studies comparing needle-based AC (alone or combined with other treatments) with pharmacotherapy, other non-pharmacotherapy or invalid groups (including placebo, waitlist and blank control) were included. The specific inclusion criteria are following:

P - Participants who were clinically diagnosed with stroke, 18 years or older, and with any degree of stroke impairment severity.

I - AC treatments alone or combined with other treatments (pharmacotherapy and non- pharmacotherapy therapies). AC treatments are specified as needle-based AC, including but not limited to manual, electro-AC, fire AC, warm AC, ear (auricular) AC, head AC, and more.

C - Pharmacotherapy, other non-pharmacotherapy, or invalid groups, including placebo, waitlist and no treatment.

O - Primary outcome was the efficacy for PSD assessed by scales measuring depressive symptoms. Secondary outcomes were the effectiveness for neurological function and quality of life.

S - Only randomized controlled trial (RCT).

Exclusion criteria were: (1) studies comparing with different types of AC; (2) total sample size was less than 30.

### Literature selection and data extraction

Two independent reviewers extracted data from selected RCTs. The basic characteristics such as first author, study title, participants (gender, age, sample size), study design (randomization, blinding), details of interventions, outcome measures, results and adverse events were abstracted and recorded into a pre-made form. Any disagreements were reviewed by a third reviewer and resolved by discussion among all reviewers.

### Risk of bias assessment

The risks of bias of included RCTs were assessed based on the Revised Cochrane Risk of Bias tool 2 [24]. Five domains were evaluated: Domain 1: Risk of bias arising from the randomization process; Domain 2: Risk of bias due to deviations from the intended interventions (effect of assignment to intervention); Domain 3: Missing outcome data; Domain 4: Risk of bias in measurement of the outcome; Domain 5: Risk of bias in selection of the reported result. Each domain was evaluated as low risk, some concerns, or high risk. The overall bias was ranked as “low risk of bias” if all domains were rated as low, or as “some concerns” if there was no high risk of bias, and all domains were rated as low or some concerns, or as “high risk of bias” if one or more domains were rated as high risk of bias. Two reviewers independently performed risk of bias assessment. Disagreements were resolved by consensus; failing that, a third reviewer made the final decision. The appraisal of acupuncture procedures was assessed by Revised Standards for Reporting Interventions in Clinical Trials of Acupuncture (STRICTA) [25].

### Assessing certainty of the evidence

The Confidence in Network Meta-Analysis (CINeMA) system, a free and open-source CINeMA software (https://cinema.ispm.unibe.ch/), was used to assess credibility of results from network meta-analyses, which is based on the Grading of Recommendations Assessment, Development and Evaluation (GRADE) s [26, 27] and simplifies the evaluation process. Six domains were evaluated, including: (a) within- study bias, (b) reporting bias, (c) indirectness, (d) imprecision, (e) heterogeneity, and (f) incoherence. Each domain was rated at 3 levels “no concerns”, “some concerns”, or “major concerns”.

### Statistical analysis

#### Pairwise meta-analysis

Risk ratios with 95% credible intervals were used for dichotomous outcomes. Mean differences or with 95% credible intervals were used for continuous outcomes. We assessed clinical and methodological heterogeneity through examination of the characteristics of the included trials. Heterogeneity across trials was assessed by *X²* and *I²* statistics. Publication bias was examined using Begg’s and funnel plot method when applicable. The contour-enhanced funnel plot was obtained as an aid to distinguish asymmetry due to publication bias.

#### Network meta-analysis

A network plot was drawn to present the geometry of the network of comparisons across trials to ensure the network meta-analyses were feasible. Trials were excluded if they were not connected by interventions.

We performed Bayesian network meta-analyses to compare the effects of different prophylactic agents because it calculates the posterior distribution of the parameters using the data to update prior information and is more common than frequentist approaches. Markov chains were used to generate samples. Model convergence was assessed using the Brooks-Gelman-Rubin plots method. Global heterogeneity was assessed on the bias of the magnitude of heterogeneity variance parameter estimated from the network meta-analyses models. All included interventions were included for synthesis of data. However, if there were treatments which could not be able to form a connected loop with other interventions, they would not be compared and analyzed in the network meta-analysis. A node-splitting method was used to examine the inconsistency between direct and indirect comparisons when a loop connecting three arms existed. The ranking probabilities for all treatments were estimated, and a treatment hierarchy using the probability of being the best treatment could be obtained. This process was performed using the Surface Under the Cumulative Ranking curve (SUCRA). We used the frequentist approach to compare stability if necessary. Statistical analysis was performed with STATA 15.0 and RStudio 3.6.0 with the package ‘GEMTC’ V.0.8.1.

## Results

### Search results

**Fig. 1:**
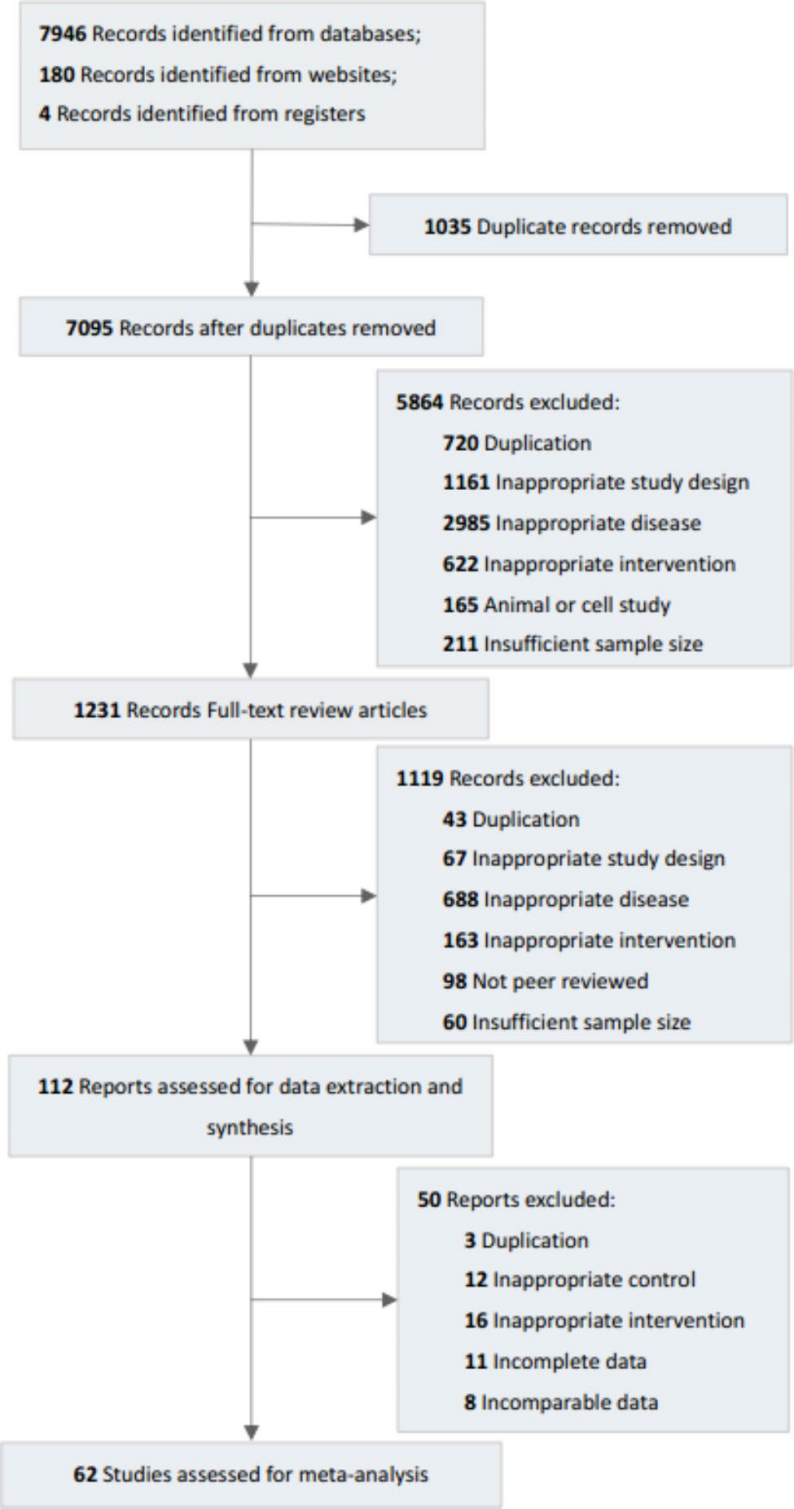
Flow diagram outlining the guideline selection process #Cochrane Library- 1032; PubMed- 2786; EMBASE- 2204; China National Knowledge Infrastructure (CNKI) -1065; Wanfang Database- 561; Chongqing VIP Database (CQVIP) – 298

Initially, 8, 130 records were identified. After screening titles and abstracts, 5,864 records were excluded and the remaining were considered potentially eligible for full-text screening. Finally, 62 studies [20, 28–89] involving 5,500participants published from 2003 to 2022 were included in this review. The flow chart of the process of study selection and studies considered for inclusion is shown in Fig. 1.

### Characteristics of included studies

The main characteristics of the included studies are shown in Table 1. Sixty-two studies involving 5,500 participants were included. All of them were from China. Apart from Usual Care (UC) (defined as controlling blood pressure and blood sugar, physical exercise or pharmacotherapy for stroke rehabilitation, and no special treatment for depression), twelve various treatments were included. Among them, acupuncture (AC) with pharmacotherapy, which is divided into AC with Western Medicine (WM) and AC with Traditional Chinese Medicine (TCM), (in this case, traditional Chinese herbal remedies), and AC with both TCM and WM. There were also five therapies comprising AC plus non-pharmacotherapy, including repetitive transcranial magnetic stimulation (RTMS), Tai Chi, Cognitive Therapy (CT), psychotherapy, and moxibustion (AM). There was one RCT exploring the treatment difference between AC with RTMS and AC alone. One study investigated the efficacy of AC combined with Tai Chi in comparison with WM. Another study evaluated AC combined with psychotherapy. Eleven studies were divided into three groups, while others were based on two groups. Twenty-eight explored the effect of AC alone in comparison with WM or UC.

**Table 1:** Description of included studies

Study	Age		Arm		Sample size	Interventions						Treatment duration	
	(Treatment /Control)				(Treatment /Control)	Group 1		Group 2		Group 3			
Xin TONG 2012^58^	58.5 ± 8.4	2		30/30			AC + WM	WM	-		8w		
Yan-xiang LIU 2010^48^	male 63.8 ± 10.0/female 59.8 ± 8.7	2		40/40			AC	UC	-		4w		
Si-qi WU 2020^64^	60.1 ± 10.7/58.3 ± 11.2	2		30/30			AC + WM	WM	-		8w		
Xiao-ling Wu 2009^65^	56 ± 9.63	2		38/36			AC + WM	WM	-		40d		
Li-jun YAO 2017^20^	65 ± 11/63 ± 12	2		30/30			AC + CT	WM	-		4w		
Lei JIANG 2011^42^	58.93 ± 3.58/59.67 ± 3.43	2		30/30			AM + WM	WM	-		6w		
Pei-yang SUN 2013^56^	58 ± 8/59 ± 9	2		30/30			AC	WM	-		4w		
Pei-yang SUN 2015^55^	59 ± 7/58 ± 8	2		33/30			AC	WM	-		4w		
Xiao CHANG 2012^28^	56.8/55.9	2		32/30			AC + TCM	WM	-		6w		
Zhong-jin ZHANG 2011^82^	56.7 ± 12.1/55.8 ± 9.7	2		40/38			AC	UC	-		3w		
Gui-bo ZHANG 2010^81^	65 ± 5/66 ± 5	2		30/30			AC + WM	WM	-		4w		
Ru ZHANG 2011^71^	50.1–71.2/49.9–68.7	2		39/39			AC + RTMS	AC	-		4w		
Lin ZHANG 2017^70^	59 ± 9/58 ± 8	2		30/30			AC	WM	-		8w		
Gang XU 2014^67^	59.4 ± 7.6/60.8 ± 6.9	2		20/18			AC + WM	WM	-		6w		
Shu-qing DAI 2010^33^	64.36 ± 5.46/65.32 ± 5.09	2		24/24			AC + TCM	WM	-		4w		
Jian ZHU 2012^85^	NR	2		20/20			AM + WM	WM	-		6w		
Yong-gang ZHU 2012^74^	67.3 ± 4.5/68.9 ± 6.1	2		21/21			AC	WM	-		8w		
Li LI 2011^45^	65 ± 13/63 ± 12	2		20/19			AC	WM	-		4w		
Hong-jie LI 2011^79^	29–60/32–63	2		23/20			AC	WM	-		6w		
Zi-ling LIN 2010^46^	52–84	2		20/20			AC	UC	-		4w		
Yun WU 2011^62^	62 ± 11/64 ± 11	2		36/36			AC	WM	-		8w		
Hai-feng JIAO 2012^44^	43–78,63.3/45–76	2		17/16			AC + WM	WM	-		4w		
Lai-qun WANG 2010^59^	40–70/40–68	2		30/30			AC + TCM	WM	-		8w		
Chang-chang YAN 2018^88^	62.1 ± 13.5/62.3 ± 13.6	2		45/45			AM + WM	WM	-		4w		
Yuan CHENG 2007^31^	72 ± 8/69 ± 7/69 ± 6	3		19/20/21			special AC	AC	UC		6w		
Yuan CHENG 2008^32^	61.7 ± 8.1/63.2 ± 7.9/62.9 ± 7.3	3		20/20/20			special AC	AC	UC		6w		
Rong-rong NIE 2011^50^	64.2 ± 9.85/63.1 ± 9.55	2		33/30			AC	WM	-		4w		
Zhi-wei SU 2010^53^	55.8 ± 6.1/54.2 ± 6.5	2		30/30			AC	WM	-		30d		
Guo-min JIANG 2007^78^	60.5	2		25/25			AC + Psych	Psych	-		4w		
Zhen-ya JIANG 2011^43^	60.32 ± 3.26/61.18 ± 2.94	2		33/33			AC + WM	WM	-		4w		
Xiao-bing ZHAO 2012^87^	56 ± 13.9/57 ± 12.8	2		45/45			AC + WM	WM	-		20d		
Ai-song GUO 2011^36^	40–65	3		32/31/32			AC	WM	AC + WM		6w		
Ai-wen CHEN 2017^29^	57 ± 11/58 ± 11	2		30/30			AC	UC	-		4w		
Lu-jie CHEN 2018^30^	62.37 ± 6.28/60.21 ± 5.78	2		30/30			AC	WM	-		4w		
Ru-hua SUI 2009^54^	62.93 ± 7.25	2		36/36			AC	UC	-		4w		
Wa GAO 2017^35^	58.12 ± 3.27	2		33/33			AC	AC + Cupping + Psych	-		10d		
Shi-le HUANG 2014^41^	61.43 ± 9.91/62.10 ± 8.11/62.77 ± 9.32	3		30/30/30			AC	WM	AC + WM		6w		
Long HUANG 2011^40^	65.22 ± 8.69/65.39 ± 11.26/63.25 ± 10.28	3		30/30/30			AC + TCM	TCM	WM		60d		
Ai-bing ZHANG 2009^80^	63 ± 11total	2		40/35			AM + TCM	WM	-		30d		
Xiao-jing DUAN 2012^77^	58.80 ± 9.60/60.22 ± 8.12	2		30/30			AC	WM	-		8w		
Feng-kui ZHU 2010^75^	NR	2		30/30			AC + WM	WM	-		4w		
Ya-hui WANG 2016^60^	66 ± 6/65 ± 7/66 ± 8	3		30/30/30			AC	UC	-		4w		
Wen-ge SUN 2012^57^	54.7 ± 2.9/54.2 ± 3.1	2		50/40			AC + TCM	UC	-		3w		
Rui-you GUO 2009^37^	65.8 ± 9.61/67.6 ± 12.43/64.5 ± 12.07	3		40/40/40			AC	WM	UC		6w		
Su-kun LIU 2006^47^	60.0 ± 9.8/59.6 ± 8.9	2		101/145			AC	WM	-		6w		
Ya-fen ZHOU 2014^84^	64 ± 15/65 ± 11	2		75/72			AC + WM	WM	-		8w		
Hui-yuan PENG 2011^51^	64.6 ± 11.3/73.7 ± 11.8	2		58/59			AC	WM	-		4w		
Wei ZHANG 2011^72^	28–80/30–83/44–85	3		52/49/49			AC + TCM	AC	TCM		45d		
Ya-qun WANG 2020^61^	70.44 ± 5.62/70.53 ± 6.44	2		52/51			AC + TCM + WM	WM	-		60d		
Rong-rong NIE 2013^49^	64 ± 10/63 ± 10/64 ± 10	3		42/41/40			AM + WM	AM	WM		4w		
Hong ZHAO 2003^86^	NR	2		50/50			AC	WM	-		8w		
Wei XIAO 2009^66^	53 ± 6/51 ± 8	2		40/40			AC + WM	WM	-		30d		
Hui-qin DING 2020^34^	54.63 ± 3.51/54.27 ± 3.43	2		61/61			AC + WM	WM	-		4w		
Jia-ping WU 2010^63^	56.2 ± 9.2/55.7 ± 9.4	2		150/150			AC	WM	-		60d		
Shu-chang SONG 2014^52^	57.25 ± 11.32/57.46 ± 11.57	2		100/100			AC	WM	-		8w		
L. ZHANG 2018^76^	63.7 ± 6.8/62.4 ± 7.6	2		45/45			AC + Tai Chi	WM	-		30d		
Zhi-en ZHOU 2020^73^	64.52 ± 1.10/65.16 ± 12.60	2		140/85			AC + TCM + WM	WM	-		12d		
Jun HE 2007^39^	53.16 ± 11.20/53.86 ± 12.34	2		180/76			AC	WM	-		30d		
Xi-jun HE 2005^38^	41–72/39–71	2		86/86			AC	WM	-		8w		
Wa CAI 2022^87^	67.8 ± 10.91/66.7 ± 11.42	2		33/32			AC	UC	-		4w		
Xiao-Jun YIN 2022^88^	58.9 ± 7.6/61.9 ± 10.0	2		30/30			AC	UC	-		4w		
Yanli YOU 2020^89^	NR	2		34/33			AC + WM	WM	-		4w		

NOTE: AC, Acupuncture; AM, Acupuncture and moxibustion; TCM, Traditional Chinese medicine; WM, Western medicine; UC, Usual care; RTMS, Repetitive transcranial magnetic stimulation; CT, Cognitive therapy; NR, not reported

### Assessment results of risk of Bias and Reporting Quality based on STRICTA

**Fig. 2:**
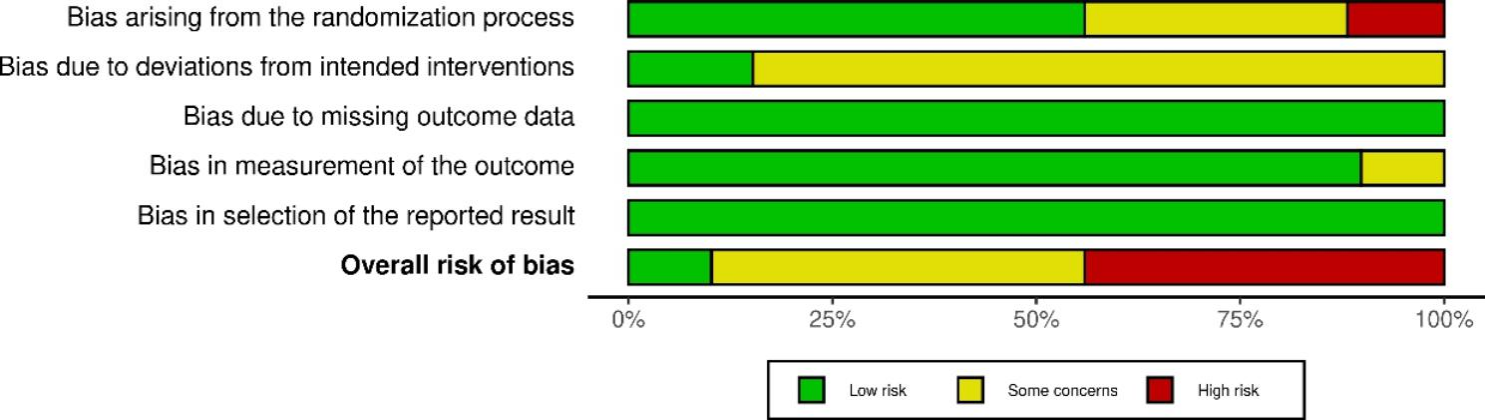
The results of ROB 2 assessment for included studies

The quality assessment results are given in Fig. 2. Seven studies (7, 11.86%) had low risk of bias arising from the randomization process (Domain 1). Nine studies (9, 15.25%) were evaluated as “low” for the risk of bias due to deviations from the intended interventions (Domain 2). In terms of overall risk of bias, 26 studies were ranked as “high”, 27 as “some concern”, and 6 as “low”. The assessment results for each domain were presented in the Supplementary Appendix 3.

**Fig. 3:**
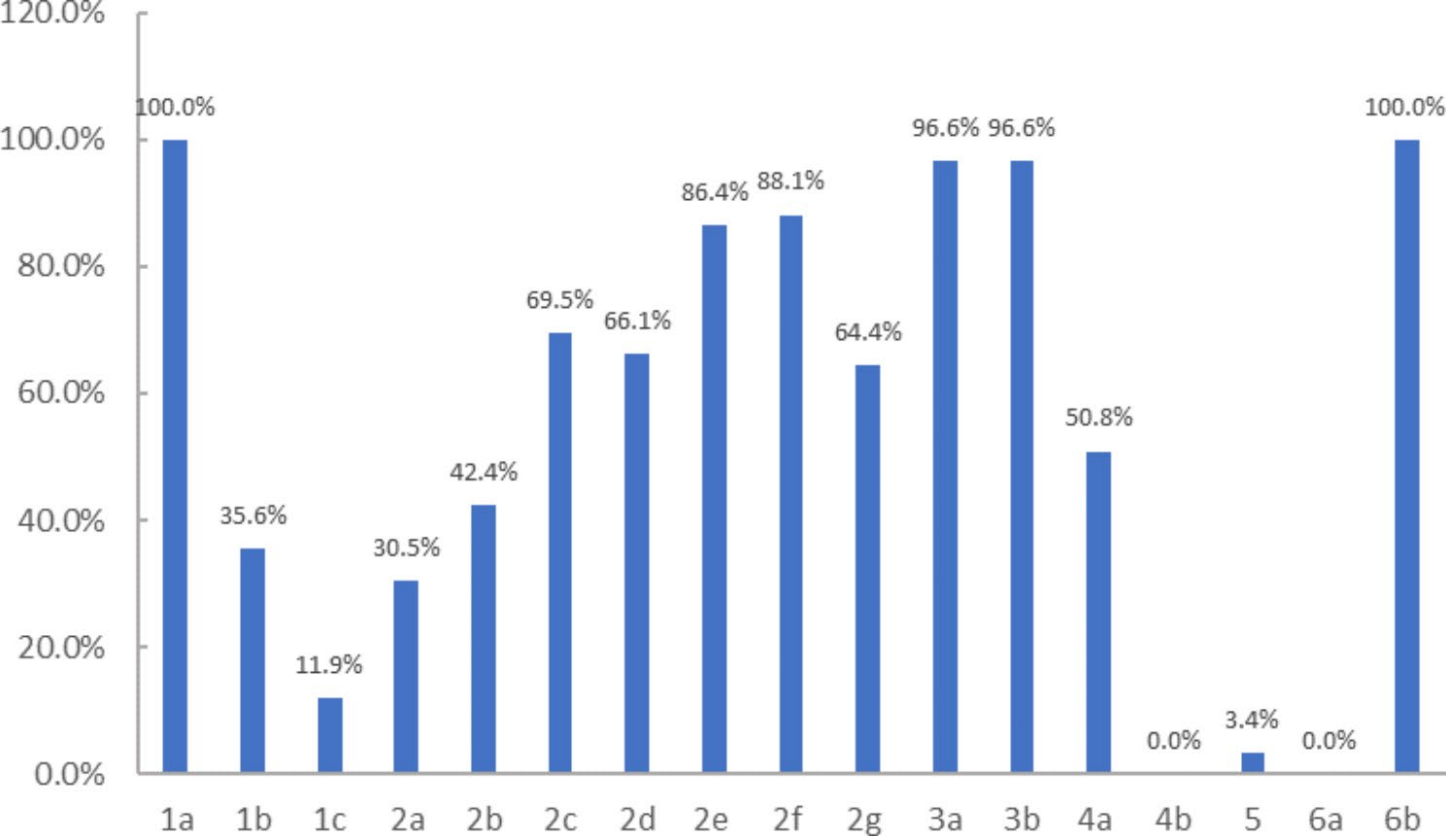
The results of STRICTA assessment for included studies Note: 1a) Style of acupuncture (e.g. Traditional Chinese Medicine, Japanese, Korean, Western medical, etc.); 1b) Reasoning for treatment provided, literature sources, and/or consensus methods, with references where appropriate; 1c) Extent to which treatment was varied; 2a) Number of needle insertions per subject per session (mean and range where relevant) ; 2b) Names (or location if no standard name) of points used (uni/bilateral) ; 2c) Depth of insertion, based on a specified unit of measurement; 2d) Response sought (e.g. de qi or muscle twitch response); 2e) Needle stimulation (e.g. manual, electrical) ; 2f) Needle retention time; 2 g) Needle type (diameter, length, and manufacturer) ; 3a) Number of treatment sessions; 3b) Frequency and duration of treatment sessions; 4a) Details of other interventions administered to the acupuncture group (e.g. moxibustion, cupping, herbs, exercises); 4b) Setting and context of treatment, including instructions to practitioners, and information and explanations to patients; 5) Description of participating acupuncturists (qualification or professional affiliation, other relevant experience) ; 6a) Rationale for the control or comparator in the context of the research question, with sources that justify this choice; 6b) Precise description of the control or comparator. If sham acupuncture or any other type of acupuncture-like control is used, provide details as for Items 1 to 3 above

The reporting quality specifically for acupuncture procedures was evaluated by STRICTA tool with 17 items. The assessment results showed that all of the studies (100%) reported the style of acupuncture (Item 1a), and 41 RCTs (66.1%) demonstrated the needle type (Item 2 g) (Fig. 3). There were 39 (66.1%) studies that presented the “response sought” (Item 2d). Only 20 (32.2%) reported the “number of needle insertions per subject per session” (Item 1c). None illustrated the “setting and context of treatment, including instructions to practitioners, and information and explanations to patients” (Item 4b) and “rationale for the control or comparator” (Item 6a). The details of the STRICTA assessment for each study are listed in Supplementary Appendix 3.

### Network meta-analysis

**Fig. 4:**
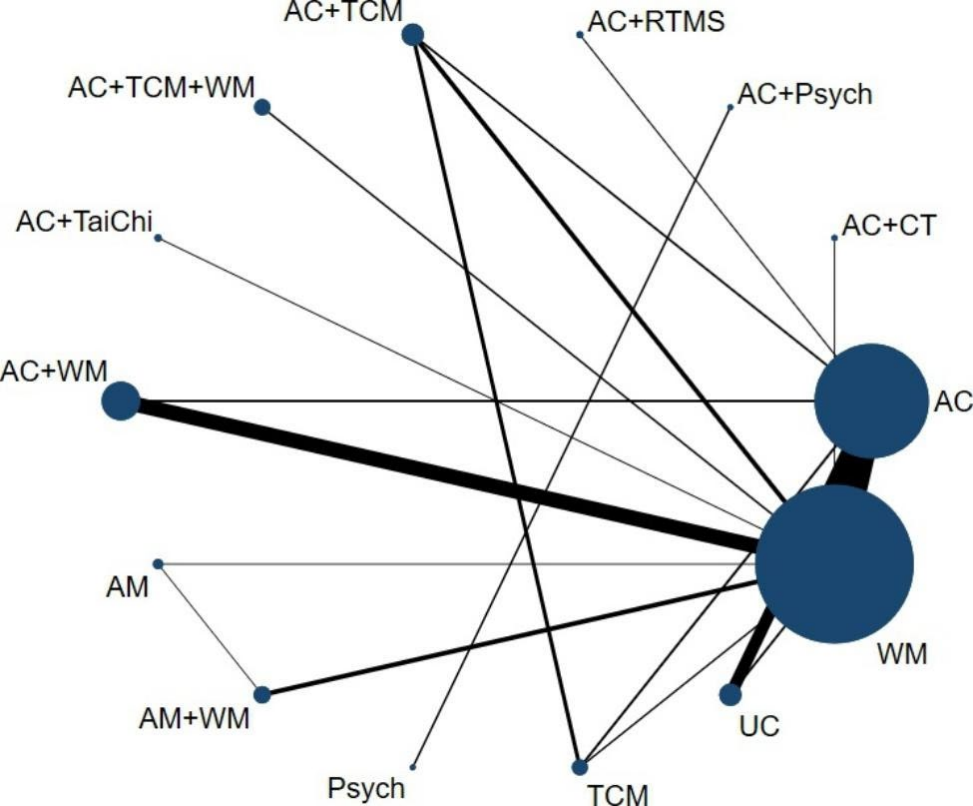
Network plot NOTE: The estimates of mean difference of treatments in the columns versus rows presented in the lower diagonal elements (while those of the row treatments vs. column treatments are presented in the upper diagonal elements). The MD of significant difference was presented in bold font. AC, Acupuncture; AM, Acupuncture and moxibustion; TCM, Traditional Chinese medicine; WM, Western medicine; UC, Usual care; RTMS, Repetitive transcranial magnetic stimulation; CT, Cognitive therapy

The network evidence plots are presented in Fig. 4. The main results were the NMA for depression. Fifty-three studies involving 4,739 patients reported changes in depression scores on the Hamilton Depression Rating Scale (HAMD), 24 for HAMD-17, 1 for HAMD-21, 28 for HAMD-24. The scores of different versions of HAMD were standardized. One study comparing the treatment of acupuncture combined with psychotherapy was excluded due to its failure to directly or indirectly connect with other interventions [78]. Six three-armed-based studies and 46 two-arm-based studies were included. A total of 110 arms were included, 42 arms for WM, 30 for AC, 12 for AC with WM, 9 for UC, 4 for AC with TCM, 4 for AM with WM, 2 for AM with TCM with WM, 2 for TCM, 1 arm for AC with RTMS, 1 for AC with Tai Chi, 1 arm for AM with TCM, 1 for AC with CT, 1 for AM. Among these studies, the largest number of studies were those comparing AC with WM (n = 20). The following comparison types of studies were AC with WM versus WM (12, 23%) and AC versus UC (9, 17%). The main results of the NMA for depression are displayed in Table 2. The results of direct comparison and indirect comparison were provided in Supplementary Appendix 4.

**Table 2:** The result of NMA for depression

AC + RTMS											
-2.90 (-12.45, 6.67)	**AC + TCM + WM**										
-2.07 (-10.64, 6.55)	0.84 (-5.55, 7.23)	**AC + TCM**									
-4.22 (-15.19, 6.81)	-1.31 (-10.65, 8.06)	-2.14 (-10.57, 6.28)	**AC + Tai Chi**								
-4.77 (-15.77, 6.29)	-1.85 (-11.21, 7.51)	-2.69 (-11.12, 5.73)	-0.55 (-11.4, 10.3)	**AC + CT**							
-3.32 (-12.71, 6.12)	-0.40 (-7.94, 7.08)	-1.25 (-6.48, 3.95)	0.90 (-8.4, 10.2)	1.43 (-7.87, 10.76)	**TCM**						
-6.39 (-16.89, 4.15)	-3.49 (-12.18, 5.25)	-4.31 (-12.03, 3.34)	-2.18 (-12.46, 8.14)	-1.62 (-11.89, 8.66)	-3.06 (-11.74, 5.59)	**AM**					
-4.29 (-12.45, 3.9)	-1.39 (-7.17, 4.39)	-2.23 (-6.32, 1.88)	-0.09 (-8.05, 7.9)	0.46 (-7.5, 8.45)	-0.98 (-6.65, 4.73)	2.09 (-5.12, 9.28)	**AC + WM**				
-6.37 (-15.16, 2.41)	-3.47 (-10.05, 3.15)	-4.31 (-9.5, 0.87)	-2.16 (-10.76, 6.44)	-1.62 (-10.21, 6.97)	-3.07 (-9.57, 3.45)	0 (-6.86, 6.84)	-2.09 (-6.52, 2.34)	**AM + WM**			
-5.26 (-13.01, 2.49)	-2.36 (-7.96, 3.25)	-3.20 (-6.93, 0.53)	-1.05 (-8.9, 6.8)	-0.51 (-8.36, 7.37)	-1.95 (-7.29, 3.41)	1.11 (-5.96, 8.16)	-0.97 (-3.61, 1.67)	1.11 (-3.09, 5.31)	**AC**		
**-8.73 (-16.64, -0.79)**	**-5.82 (-11.17, -0.46)**	**-6.67 (-10.15, -3.18)**	-4.52 (-12.18, 3.15)	-3.97 (-11.64, 3.71)	**-5.42 (-10.67, -0.14)**	-2.36 (-9.21, 4.51)	**-4.44 (-6.62, -2.26)**	-2.35 (-6.19, 1.49)	**-3.47 (-5.15, -1.78)**	**WM**	
**-13.30 (-21.47, -5.13)**	**-10.40 (-16.55, -4.27)**	**-11.23 (-15.77, -6.74)**	**-9.09 (-17.34, -0.85)**	**-8.54 (-16.80, -0.28)**	**-9.98 (-15.91, -4.06)**	**-6.91 (-14.45, -0.12)**	**-9.00 (-12.66, -5.36)**	**-6.91 (-11.83, -2.05)**	**-8.03 (-10.63, -5.45)**	**-4.56 (-7.61, -1.55)**	**UC**

NOTE: The estimates of mean difference of treatments in the columns versus rows presented in the lower diagonal elements (while those of the row treatments vs. column treatments are presented in the upper diagonal elements). The MD of significant difference was presented in bold font. AC, Acupuncture; AM, Acupuncture and moxibustion; TCM, Traditional Chinese medicine; WM, Western medicine; UC, Usual care; RTMS, Repetitive transcranial magnetic stimulation; CT, Cognitive therapy

On the whole, interventions administering combined therapies were more effective in comparison with those using single therapy. The results of NMA showed that compared with WM alone, the administration of AC with RTMS were superior in alleviating depression symptoms (MD: -8.73, 95% CI: -16.64, -0.79). There were similar results when comparing AC with TCM and WM, AC with TCM, TCM alone, AC with WM, and AC alone. Compared to UC, AC alone or in combination with other interventions could lower HAMD scores (*AC with RTMS*: MD, -13.30, 95% CI: -21.47, -5.13; *AC with TCM with WM*: MD, -10.40, 95% CI: -16.55, -4.27; *AC with TCM*: MD, -11.35, 95% CI: -15.77, -6.74; *AC with Tai Chi*: MD, -9.09, 95% CI: -17.34, -0.85; *AC with CT*: MD, -8.54, 95% CI: -16.80, -0.28; *TCM*: MD, -9.98, 95% CI: -15.91, -4.06; *AM*: MD, -6.91, 95% CI: -14.45, -0.12; *AC with WM*: MD, -9.00, 95% CI: -12.66, -5.36; *AM with WM*: MD, -6.91, 95% CI: -11.83, -2.05; *AC*: MD, -8.03, 95% CI: -10.63, -5.45; *WM*: MD, -4.56, 95% CI: -7.61, -1.55). However, no significant difference was found among AC, WM, and TCM with AC plus any other treatment (AC with RTMS, AC with TCM, AC with TCM with WM, AC with Tai Chi, AC with WM, AC with CT, AM and AM with WM).

The analysis of heterogeneity showed the *I*^*2*^ of direct comparison was 97.66%, and the global *I*^*2*^ was 97.38%. The *I*^*2*^ of each comparison group was seen in Supplementary Appendix 5. Examination of consistency with the node-splitting analysis approach indicated that there was no significant inconsistency (*P* > 0.05) (Supplementary Appendix 6).

Table 3 presents the values of SUCRA, the hierarchy of thirteen treatments. According to SUCRA, AC plus RTMS had the highest probability of improving depressive symptoms with a probability of 49.39%. The next were AC with TCM with WM (11.11%), AC with TCM (10.61%), AC with Tai Chi (10.34%), which were very close. The probability of AC with CT and TCM were 8.13% and 7.32% respectively. The figure for SUCRA is attached in Supplementary Appendix 7.

**Table 3:** Ranking probability of different interventions

	Treatments	Cumulative Probability
1	AC + RTMS	49.39%
2	AC + TCM + WM	11.11%
3	AC + TCM	10.61%
4	AC + Tai Chi	10.34%
5	AC + CT	8.13%
6	TCM	7.32%
7	AM	2.53%
8	AC + WM	0.37%
9	AM + WM	0.16%
10	AC	0.01%
11	WM	0.00%
12	UC	0.00%

NOTE: AC, Acupuncture; AM, Acupuncture and moxibustion; TCM, Traditional Chinese medicine; WM, Western medicine; UC, Usual care; RTMS, Repetitive transcranial magnetic stimulation; CT, Cognitive therapy

### Pairwise meta-analysis

**Fig. 5:**
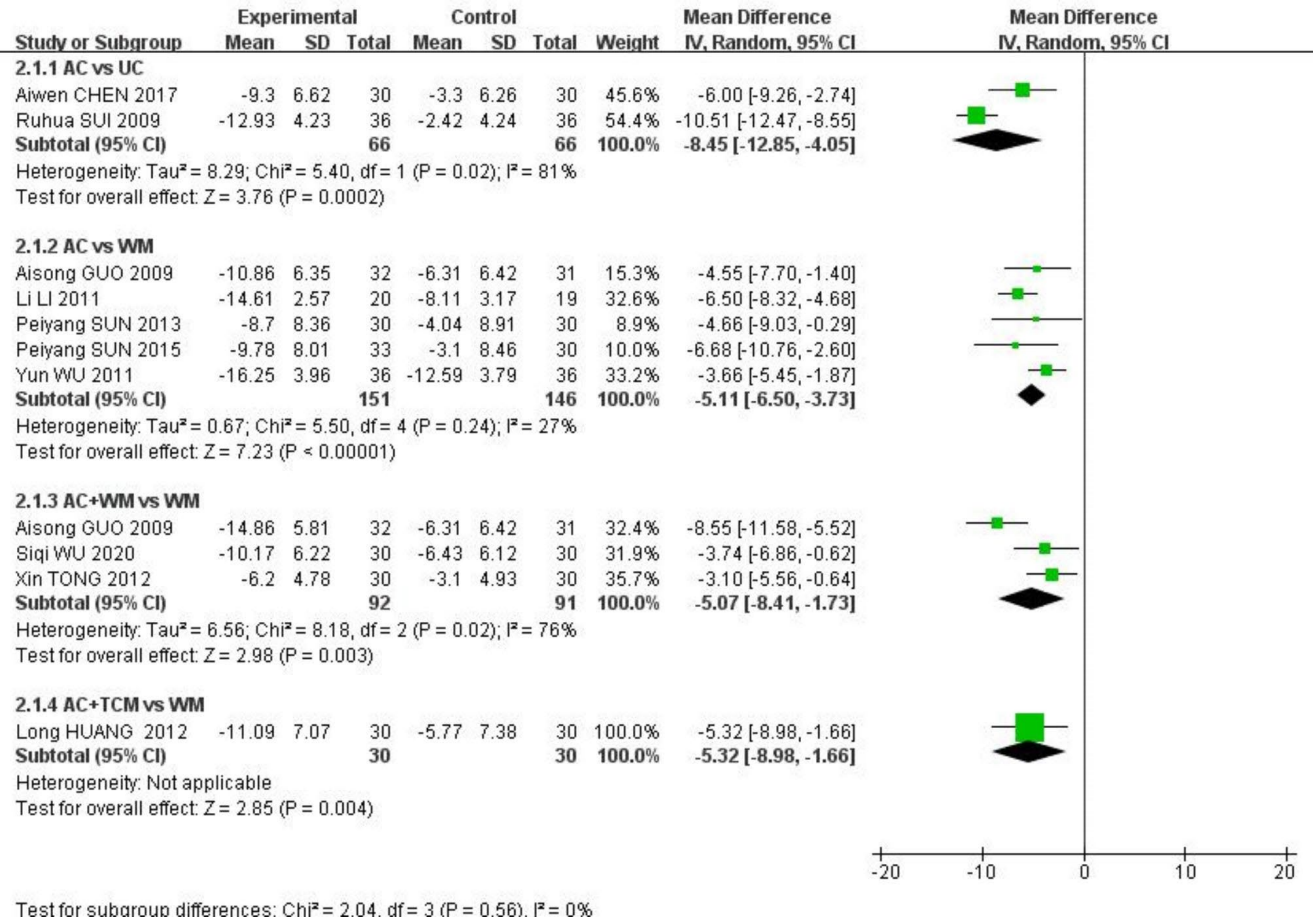
The forest plot for neurological impairment

Ten studies [29, 36, 40, 45, 54–56, 58, 62, 64] used the Modified Edinburgh-Scandinavian Stroke Scale (MESSS) to measure neurological impairment. As shown in Fig. 5, the results of pairwise meta-analysis showed that AC was significantly associated with better neurological function improvement than UC or WM (MD, -8.45, 95% CI: -12.85, -4.05; MD, -5.11, 95% CI: -6.50, -3.73, respectively). Similarly, AC with WM and AC with TCM were superior to WM (MD, -5.07, 95% CI: -8.41, -1.73; MD, -5.32, 95% CI: -8.89, -1.66, respectively).

### The confidence assessment result of NMA

The CINeMA system (https://cinema.ispm.unibe.ch/) was used to classify the confidence in the results of NMA, which six domains was evaluated, including: (a) within- study bias, (b) reporting bias, (c) indirectness, (d) imprecision, (e) heterogeneity, and (f) incoherence. One comparison (AC versus AC with TCM) was ranked “high”; eight were “moderate”; eight were “low”. The details are presented in Table 4.

**Table 4:** The result of certainty assessment for depression scores

Comparison	Number of studies	Within-study bias	Reporting bias	Indirectness	Imprecision	Heterogeneity	Incoherence	Confidence rating
AC vs. AC with RTMS	1	Major concerns	No concerns	No concerns	Some concerns	Some concerns	No concerns	Low
**AC vs. AC with TCM**	1	No concerns	No concerns	No concerns	Some concerns	No concerns	No concerns	High
**AC vs. AC with WM**	2	Some concerns	No concerns	No concerns	No concerns	Major concerns	No concerns	Low
**AC vs. TCM**	1	No concerns	No concerns	No concerns	Some concerns	Some concerns	No concerns	Moderate
AC vs. UC	9	Some concerns	No concerns	No concerns	No concerns	Some concerns	No concerns	Moderate
**AC vs. WM**	21	Some concerns	No concerns	No concerns	No concerns	Some concerns	No concerns	Moderate
AC with CT vs. WM	1	Some concerns	No concerns	No concerns	Some concerns	Some concerns	No concerns	Moderate
AC with TCM vs. TCM	2	Major concerns	No concerns	No concerns	Some concerns	Some concerns	No concerns	Low
**AC with TCM vs. WM**	4	Major concerns	No concerns	No concerns	No concerns	Some concerns	No concerns	Low
AC with TCM with WM vs. WM	2	Some concerns	No concerns	No concerns	Some concerns	No concerns	No concerns	Moderate
AC with Tai Chi vs. WM	1	Major concerns	No concerns	No concerns	Some concerns	Some concerns	No concerns	Low
AC with WM vs. WM	11	Some concerns	No concerns	No concerns	No concerns	Some concerns	Some concerns	Moderate
AM vs. AM with WM	1	Some concerns	No concerns	No concerns	Major concerns	No concerns	No concerns	Low
AM vs. WM	1	Some concerns	No concerns	No concerns	Some concerns	Some concerns	No concerns	Moderate
AM with WM vs. WM	4	Some concerns	No concerns	No concerns	No concerns	Major concerns	No concerns	Low
**TCM vs. WM**	1	Major concerns	No concerns	No concerns	Some concerns	No concerns	No concerns	Low
**UC vs. WM**	1	Some concerns	No concerns	No concerns	No concerns	Some concerns	No concerns	Moderate

NOTE: AC, Acupuncture; AM, Acupuncture and moxibustion; TCM, Traditional Chinese medicine; WM, Western medicine; UC, Usual care; RTMS, Repetitive transcranial magnetic stimulation; CT, Cognitive therapy

## Discussion

This network meta-analysis, involving sixty-two studies, found that compared with WM, AC alone or in combination with RTMS, TCM, WM, or TCM with WM seemed to be more effective in improving depression symptoms of PSD. When compared with UC, AC alone or combined with any other therapy (including AC with RTMS, AC with TCM, AC with TCM and WM, AC with Tai Chi, AC with WM, AC with CT, AM, and AM with WM) was superior. Additionally, the pairwise meta-analysis showed that combined therapies of AC with WM, AC with TCM, and AC alone were significantly associated with better neurological function improvement in contrast to UC or WM.

The results of SUCRA showed that AC with RTMS had the highest probability of being the most effective therapy to alleviate depressive symptoms. It is reported that RTMS, as a non-invasive approach, could induce excitability changes in the motor cortex via a wire coil generating a magnetic field that passes through the scalp. However, as a novel approach to treat depression, the physiological mechanisms of RTMS after-effects are yet to be clear [90]. The updated evidence-based guidelines by Lefaucheur et al. demonstrated that RTMS has a significant effect in several psychiatric diseases. It is also highly recommended (Level A) for major depression, [91]. which was consistent with our findings. A randomised trial explored the effectiveness of intermittent theta burst stimulation versus high-frequency RTMS (10 Hz) for patients with treatment-resistant depression. It found that of intermittent theta burst stimulation was non-inferior to 10 Hz RTMS for the treatment of depression, which both treatments had low numbers of side-effects, safety, and tolerability profiles [92, 93]. Nonetheless, it needs to be interpreted with caution because there was only one eligible study included which compared AC alone and AC with RTMS in this network meta-analysis. More relevant research is needed to confirm the true function and safety of RTMS for patients with PSD.

In this study, various combined therapies were evaluated, including AC with RTMS, AM with TCM, AC with Tai Chi, AC with TCM with WM, AC with TCM, AC with CT, AC with WM, AM with WM. We first explored the difference in effectiveness for PSD between these therapies using the network meta-analysis approach, which synthesized the direct comparison evidence and indirect comparison evidence. However, some comparisons contained only a few eligible studies, such as those on AC with RTMS versus AC, AC with CT versus WM, and AC with Tai Chi versus WM. Therefore, it is suggested that future research could focus on the effectiveness of these different comparisons for patients with PSD. We will also conduct an update if further studies are published.

Moreover, it is noticeable that the treatment duration among included studies was various and ranged from 2 weeks to 8 weeks, which could be signaled as a potential modifier. Therefore, we explored pairwise meta-analyses for all direct comparisons (Supplementary Appendix 8). A total of seventeen pairwise meta-analysis were conducted, including AC vs. WM (20 studies), AC with WM vs. WM (11 studies), AC vs. UC (7 studies), AC with TCM vs. WM (4 studies), AM with WM vs. WM (4 studies), AC vs. AC with WM (2 studies), AC with TCM vs. TCM (2 studies), AC with TCM with WM vs. WM (2), AC vs. AC with RTMS (1 studies), AC vs. AC with TCM (1 studies), AC vs. TCM ((1 studies), AC with CT vs. WM (1 studies), AC with TJ vs. WM (1 studies), AM vs. AM with WM (1 studies), AM vs. WM (1 studies), TCM vs. WM (1 studies), and UC vs. WM (1 studies). The six subgroup analysis was explored, in which the treatment duration was divided into short-term group (≤ 4 weeks) or long-term group (>4 weeks). However, it failed to show subgroup effects regarding treatment duration.

One published network meta-analysis by Hang et al. compared the effect of different acupuncture approaches for specific parts in treating patients with PSD [94]. Twelve acupuncture therapies were included in their study, and they found that scalp acupuncture plus conventional acupuncture was the most effective method based on the ranking probability. For this study, we put more focus on the effectiveness of acupuncture combined with other therapies for PSD. Due to the complexity in the nature of acupuncture treatments and their procedures, we were not able to conduct a relevant quantitative subgroup analysis. Therefore, we could only differentiate the types of acupuncture by their main characteristics, which induced huge heterogeneity among acupuncture treatments. The details were presented in Supplementary Appendix 9. The most frequently used acupoints included Bai-Hui, Shen-Ting, and Nei-Guan.

This is the first review to compare the effectiveness of acupuncture with other therapies for PSD using a network meta-analysis, which may provide novel and useful guidance for clinicians and readers. However, there are still several limitations. Firstly, although we conducted a systematic search for eligible studies, only Chinese studies were eligible and subsequently included. Secondly, the newest assessment tool of ROB 2 was used to evaluate the quality of included RCTs, and the assessment results indicated that the overall quality of RCTs was not high. Therefore, more high-quality RCTs are needed.

## Conclusions

In our review, we provide an overview of the current research evidence on the effectiveness of AC alone or in combination with other therapies for the treatment of PSD. Twelve different treatments are included. Among them, there are five therapies of AC plus non-pharmacotherapy (RTMS, Tai Chi, CT, psychotherapy, moxibustion), while AC plus pharmacotherapy treatments also include AC with WM and AC with TCM. The results of NMA indicate that combined therapies, including AC with RTMS, AC with TCM, AC with TCM with WM, AC with WM, and AC alone may be more effective in alleviating depression symptoms as compared with WM. With the highest probability, AC with RTMS seems to be the most effective with the highest probability in treating PSD. Nonetheless, more high-quality studies are needed to provide sufficient evidence.

## Electronic supplementary material

Below is the link to the electronic supplementary material.

Supplementary material 1. Reporting checklist (PRISMA-NMA)

Supplementary material 2. Search strategies in PubMed

Supplementary material 3. The results of ROB and STRICTA assessment for included studies

Supplementary material 4. The results of direct and indirect comparison

Supplementary material 5. The figure of results of analysis of heterogeneity

Supplementary material 6. The figure of node-splitting analysis of inconsistency

Supplementary material 7. The figure of SUCRA

Supplementary material 8. The forest plots for all direct pairwise meta-analysis

Supplementary material 9. The main point of acupuncture of included studies

## Data Availability

All data generated or analyzed during this study are included in this published article and its supplementary information files.

## Abbreviations

AC: Acupuncture
AM: Acupuncture and moxibustion
TCM: Traditional Chinese medicine
WM: Western medicine
UC: Usual care
RTMS: Repetitive transcranial magnetic stimulation
CT: Cognitive therapy
PSD: Post-stroke depression
RCTs: Randomized clinical trials
NMA: Network meta-analysis
SUCRA: Surface Under the Cumulative Ranking curve
HAMD: Hamilton Depression Rating Scale
