# Supplementary material for: Acupuncture for post-stroke depression: a systematic review and network meta-analysis
Source: BMC Psychiatry. 2023 May 4;23:314. doi: 10.1186/s12888-023-04749-1 (PMC10161596; doi:10.1186/s12888-023-04749-1)
Supplement: Supplementary file 2 — Supplementary material 2. Search strategies in PubMed [file 12888_2023_4749_MOESM2_ESM.docx]

**Supplementary Appendix 3.**

**Table 1. The results of ROB assessment for included studies.**

| **ID** | **Study** | **Domain 1** | **Domain 2** | **Domain 3** | **Domain 4** | **Domain 5** | **Overall** |
| --- | --- | --- | --- | --- | --- | --- | --- |
| 5001 | Xin TONG 2012 | Some concerns | Some concerns | Low | Low | Low | High |
| 5076 | Yanxiang LIU 2010 | Some concerns | Some concerns | Low | Low | Low | High |
| 5245 | Siqi WU 2020 | Low | Some concerns | Low | Low | Low | Some concerns |
| 5250 | Xiaoling Wu 2009 | Some concerns | Some concerns | Low | Low | Low | High |
| 5357 | Lijun YAO 2017 | Low | Some concerns | Low | Low | Low | Some concerns |
| 5370 | Lei JIANG 2011 | Some concerns | Some concerns | Low | Low | Low | High |
| 5391 | Peiyang SUN 2013 | Low | Some concerns | Low | Low | Low | Some concerns |
| 5390 | Peiyang SUN 2015 | Low | Some concerns | Low | Low | Low | Some concerns |
| 5514 | Xiao CHNAG 2012 | Low | Some concerns | Low | Low | Low | Some concerns |
| 5543 | Zhongjin ZHANG 2011 | Some concerns | Some concerns | Low | Low | Low | High |
| 5597 | Guibo ZHANG 2010 | Some concerns | Some concerns | Low | Some concerns | Low | High |
| 5624 | Ru ZHANG 2011 | Some concerns | Some concerns | Low | Low | Low | High |
| 5668 | Lin ZHANG 2017 | Low | Low | Low | Low | Low | Low |
| 5700 | Gang XU 2014 | Low | Some concerns | Low | Low | Low | Some concerns |
| 5738 | Shuqing DAI 2010 | Low | Some concerns | Low | Low | Low | Some concerns |
| 5794 | Jian ZHU 2012 | Some concerns | Some concerns | Low | Low | Low | High |
| 5805 | Yonggang ZHU 2012 | High | Some concerns | Low | Low | Low | High |
| 5995 | Li LI 2011 | Some concerns | Some concerns | Low | Low | Low | High |
| 6012 | Hongjie LI 2011 | Low | Some concerns | Low | Low | Low | Some concerns |
| 6069 | Ziling LIN 2010 | Some concerns | Low | Low | Low | Low | Some concerns |
| 6242 | Yun WU 2011 | Low | Some concerns | Low | Low | Low | Some concerns |
| 6247 | Haifeng JIAO 2012 | Some concerns | Some concerns | Low | Low | Low | High |
| 6372 | Laiqun WANG 2010 | Some concerns | Some concerns | Low | Low | Low | High |
| 6304 | Changchang YAN 2018 | Low | Some concerns | Low | Low | Low | Some concerns |
| 6545 | Yuan CHENG 2007 | Low | Some concerns | Low | Low | Low | Some concerns |
| 6546 | Yuan CHENG 2018 | Low | Low | Low | Low | Low | Low |
| 6588 | Rongrong NIE 2011 | Low | Some concerns | Low | Low | Low | Some concerns |
| 6662 | Zhiwei SU 2010 | Low | Some concerns | Low | Some concerns | Low | High |
| 6703 | Guomin JIANG 2010 | Some concerns | Some concerns | Low | Low | Low | High |
| 6705 | Zhenya JIANG 2011 | Low | Some concerns | Low | Low | Low | Some concerns |
| 6839 | Xiaobing ZHAO 2012 | Some concerns | Some concerns | Low | Some concerns | Low | High |
| 6942 | Aisong GUO 2009 | Some concerns | Low | Low | Low | Low | Some concerns |
| 7043 | Aiwen CHEN 2017 | Low | Some concerns | Low | Low | Low | Some concerns |
| 7083 | Lujie CHEN 2009 | High | Some concerns | Low | Low | Low | High |
| 7089 | Ruhua SUI 2009 | High | Some concerns | Low | Low | Low | High |
| 7182 | Wa GAO 2017 | Low | Some concerns | Low | Some concerns | Low | High |
| 7213 | Shile HUANG 2007 | Low | Low | Low | Low | Low | Low |
| 7271 | Long HUANG 2012 | Some concerns | Some concerns | Low | Low | Low | High |
| 5604 | Aibing ZHANG 2009 | Some concerns | Some concerns | Low | Some concerns | Low | High |
| 6169 | Xiaojing DUAN 2012 | Low | Some concerns | Low | Low | Low | Some concerns |
| 6522 | Fengkui ZHU 2010 | Some concerns | Some concerns | Low | Some concerns | Low | High |
| 6279 | Yahui WANG 2016 | Low | Some concerns | Low | Low | Low | Some concerns |
| 5400 | Wenge SUN 2012 | Low | Some concerns | Low | Low | Low | Some concerns |
| 1095 | Ruiyou GUO 2009 | Low | Low | Low | Low | Low | Low |
| 5113 | Sukun LIU 2006 | Low | Some concerns | Low | Low | Low | Some concerns |
| 5273 | Yafen ZHOU 2014 | Low | Low | Low | Low | Low | Low |
| 5690 | Huiyuan PENG 2011 | Low | Some concerns | Low | Low | Low | Some concerns |
| 5677 | Wei ZHANG 2011 | Low | Low | Low | Low | Low | Low |
| 6270 | Yaqun WANG 2020 | Low | Some concerns | Low | Low | Low | Some concerns |
| 2649 | Rongrong NIE 2013 | Low | Some concerns | Low | Low | Low | Some concerns |
| 6862 | Hong ZHAO 2003 | Low | Some concerns | Low | Low | Low | Some concerns |
| 6615 | Wei XIAO 2009 | High | Some concerns | Low | Low | Low | High |
| 4926 | Huiqin DING 2020 | Low | Some concerns | Low | Low | Low | Some concerns |
| 5242 | Jiaping WU 2010 | High | Some concerns | Low | Low | Low | High |
| 3169 | Shuchang SONG 2014 | Low | Some concerns | Low | Low | Low | Some concerns |
| 4457 | L. ZHANG 2018 | High | Some concerns | Low | Low | Low | High |
| 5298 | Zhien ZHOU 2020 | Low | Some concerns | Low | Low | Low | Some concerns |
| 1175 | Jun HE 2007 | High | Low | Low | Low | Low | High |
| 1193 | Xijun HE 2005 | Some concerns | Some concerns | Low | Low | Low | High |

Note: Domain 1: Risk of bias arising from the randomization process; Domain 2: Risk of bias due to deviations from the intended interventions (effect of assignment to intervention); Domain 3: Missing outcome data; Domain 4: Risk of bias in measurement of the outcome; Domain 5: Risk of bias in selection of the reported result.

**Table 2. The results of STRICTA assessment for included studies.**

| **ID** | **Study** | **1 Acupuncture rationale** | | | **2 Details of needling** | | | | | | | **3 Treatment regimen** | | **4 Other components of treatment** | | **5 Practitioner background** | **6 Control or comparator interventions** | |
| --- | --- | --- | --- | --- | --- | --- | --- | --- | --- | --- | --- | --- | --- | --- | --- | --- | --- | --- |
|  |  | 1a | 1b | 1c | 2a | 2b | 2c | 2d | 2e | 2f | 2g | 3a | 3b | 4a | 4b | 5 | 6a | 6b |
| 5001 | Xin TONG 2012 | Y | N | N | Y | Y | N | Y | N | Y | Y | Y | Y | Y | N | N | N | Y |
| 5076 | Yanxiang LIU 2010 | Y | N | N | Y | Y | Y | Y | N | Y | Y | Y | Y | Y | N | N | N | Y |
| 5245 | Siqi WU 2020 | Y | Y | N | N | Y | Y | Y | Y | Y | Y | Y | Y | Y | N | N | N | Y |
| 5250 | Xiaoling Wu 2009 | Y | N | N | N | Y | Y | N | Y | Y | Y | Y | Y | Y | N | N | N | Y |
| 5357 | Lijun YAO 2017 | Y | Y | Y | N | Y | Y | Y | Y | Y | Y | Y | Y | Y | N | N | N | Y |
| 5370 | Lei JIANG 2011 | Y | N | N | Y | Y | Y | Y | Y | Y | Y | Y | Y | Y | N | N | N | Y |
| 5391 | Peiyang SUN 2013 | Y | Y | N | Y | Y | Y | Y | Y | Y | Y | Y | Y | N | N | N | N | Y |
| 5390 | Peiyang SUN 2015 | Y | Y | Y | Y | N | Y | Y | Y | Y | Y | Y | Y | N | N | N | N | Y |
| 5514 | Xiao CHNAG 2012 | Y | N | N | Y | Y | N | N | Y | Y | N | Y | Y | Y | N | N | N | Y |
| 5543 | Zhongjin ZHANG 2011 | Y | N | N | N | N | N | N | N | Y | N | Y | Y | Y | N | N | N | Y |
| 5597 | Guibo ZHANG 2010 | Y | N | N | Y | N | Y | N | Y | N | N | Y | Y | N | N | N | N | Y |
| 5624 | Ru ZHANG 2011 | Y | N | Y | N | Y | N | Y | Y | Y | Y | Y | Y | Y | N | N | N | Y |
| 5668 | Lin ZHANG 2017 | Y | Y | Y | N | N | Y | Y | Y | Y | Y | Y | Y | Y | N | N | N | Y |
| 5700 | Gang XU 2014 | Y | N | N | N | N | N | N | N | N | N | Y | Y | N | N | N | N | Y |
| 5738 | Shuqing DAI 2010 | Y | N | N | N | N | Y | N | Y | Y | N | Y | Y | Y | N | N | N | Y |
| 5794 | Jian ZHU 2012 | Y | N | Y | Y | Y | N | N | N | N | N | Y | N | N | N | N | N | Y |
| 5805 | Yonggang ZHU 2012 | Y | N | N | Y | Y | N | Y | Y | Y | Y | Y | Y | Y | N | N | N | Y |
| 5995 | Li LI 2011 | Y | Y | N | N | Y | Y | Y | Y | Y | N | Y | Y | N | N | N | N | Y |
| 6012 | Hongjie LI 2011 | Y | Y | Y | N | N | Y | Y | Y | Y | Y | Y | Y | N | N | N | N | Y |
| 6069 | Ziling LIN 2010 | Y | N | N | N | N | N | Y | Y | Y | N | Y | Y | Y | N | N | N | Y |
| 6242 | Yun WU 2011 | Y | N | N | N | Y | Y | Y | Y | Y | Y | Y | Y | N | N | N | N | Y |
| 6247 | Haifeng JIAO 2012 | Y | N | N | N | N | Y | Y | Y | Y | Y | Y | Y | N | N | N | N | Y |
| 6372 | Laiqun WANG 2010 | Y | N | N | N | N | Y | N | Y | Y | N | Y | Y | Y | N | N | N | Y |
| 6304 | Changchang YAN 2018 | Y | N | N | N | N | Y | Y | Y | Y | Y | Y | Y | Y | N | Y | N | Y |
| 6545 | Yuan CHENG 2007 | Y | Y | N | N | Y | Y | Y | Y | Y | Y | Y | Y | N | N | N | N | Y |
| 6546 | Yuan CHENG 2018 | Y | Y | N | N | Y | Y | Y | Y | Y | Y | Y | Y | N | N | N | N | Y |
| 6588 | Rongrong NIE 2011 | Y | N | N | N | N | Y | Y | Y | Y | Y | Y | Y | Y | N | N | N | Y |
| 6662 | Zhiwei SU 2010 | Y | N | N | Y | Y | N | Y | Y | Y | Y | Y | Y | N | N | N | N | Y |
| 6703 | Guomin JIANG 2010 | Y | Y | N | N | N | N | N | Y | Y | Y | Y | Y | N | N | N | N | Y |
| 6705 | Zhenya JIANG 2011 | Y | N | N | Y | N | Y | Y | Y | Y | Y | Y | Y | N | N | N | N | Y |
| 6839 | Xiaobing ZHAO 2012 | Y | Y | N | Y | N | N | N | Y | Y | N | Y | Y | N | N | N | N | Y |
| 6942 | Aisong GUO 2009 | Y | N | N | Y | N | N | N | Y | Y | Y | Y | Y | N | N | N | N | Y |
| 7043 | Aiwen CHEN 2017 | Y | Y | N | Y | N | Y | Y | Y | Y | Y | Y | Y | N | N | N | N | Y |
| 7083 | Lujie CHEN 2009 | Y | Y | N | N | Y | Y | Y | Y | Y | Y | Y | Y | N | N | N | N | Y |
| 7089 | Ruhua SUI 2009 | Y | N | N | N | N | Y | Y | Y | Y | Y | Y | Y | N | N | N | N | Y |
| 7182 | Wa GAO 2017 | Y | Y | N | N | N | N | N | N | N | N | Y | Y | N | N | N | N | Y |
| 7213 | Shile HUANG 2007 | Y | N | N | N | Y | Y | N | Y | Y | Y | Y | Y | N | N | N | N | Y |
| 7271 | Long HUANG 2012 | Y | N | N | N | N | Y | Y | Y | Y | N | Y | Y | Y | N | N | N | Y |
| 5604 | Aibing ZHANG 2009 | Y | N | N | N | N | Y | N | Y | Y | N | Y | Y | Y | N | N | N | Y |
| 6169 | Xiaojing DUAN 2012 | Y | N | N | N | N | N | Y | Y | Y | N | Y | Y | N | N | N | N | Y |
| 6522 | Fengkui ZHU 2010 | Y | N | N | Y | Y | Y | Y | Y | Y | Y | N | N | N | N | N | N | Y |
| 6279 | Yahui WANG 2016 | Y | N | N | N | Y | Y | Y | Y | Y | Y | Y | Y | Y | N | N | N | Y |
| 5400 | Wenge SUN 2012 | Y | N | N | N | N | Y | N | Y | Y | Y | Y | Y | Y | N | N | N | Y |
| 1095 | Ruiyou GUO 2009 | Y | N | N | N | N | N | Y | Y | Y | Y | Y | Y | Y | N | N | N | Y |
| 5113 | Sukun LIU 2006 | Y | N | N | N | N | N | N | N | Y | N | Y | Y | N | N | N | N | Y |
| 5273 | Yafen ZHOU 2014 | Y | Y | N | N | Y | Y | N | Y | Y | Y | Y | Y | Y | N | Y | N | Y |
| 5690 | Huiyuan PENG 2011 | Y | N | N | N | Y | Y | Y | Y | Y | N | Y | Y | Y | N | N | N | Y |
| 5677 | Wei ZHANG 2011 | Y | N | N | N | N | N | N | N | N | N | Y | Y | Y | N | N | N | Y |
| 6270 | Yaqun WANG 2020 | Y | N | N | N | N | Y | Y | Y | N | Y | Y | Y | Y | N | N | N | Y |
| 2649 | Rongrong NIE 2013 | Y | N | N | N | N | Y | Y | Y | Y | Y | Y | Y | Y | N | N | N | Y |
| 6862 | Hong ZHAO 2003 | Y | N | N | N | N | N | N | Y | N | N | N | Y | N | N | N | N | Y |
| 6615 | Wei XIAO 2009 | Y | Y | N | N | Y | Y | Y | Y | Y | Y | Y | Y | N | N | N | N | Y |
| 4926 | Huiqin DING 2020 | Y | Y | N | N | N | Y | Y | Y | Y | Y | Y | Y | N | N | N | N | Y |
| 5242 | Jiaping WU 2010 | Y | Y | N | Y | N | Y | Y | Y | Y | N | Y | Y | Y | N | N | N | Y |
| 3169 | Shuchang SONG 2014 | Y | Y | N | N | N | Y | Y | Y | Y | Y | Y | Y | N | N | N | N | Y |
| 4457 | L. ZHANG 2018 | Y | N | N | N | Y | Y | Y | Y | Y | Y | Y | Y | Y | N | N | N | Y |
| 5298 | Zhien ZHOU 2020 | Y | Y | Y | Y | N | Y | Y | Y | Y | Y | Y | Y | Y | N | N | N | Y |
| 1175 | Jun HE 2007 | Y | Y | N | N | Y | Y | Y | Y | Y | N | Y | Y | N | N | N | N | Y |
| 1193 | Xijun HE 2005 | Y | N | N | Y | N | Y | N | Y | Y | N | Y | Y | Y | N | N | N | Y |

Note: 1a) Style of acupuncture (e.g. Traditional Chinese Medicine, Japanese, Korean, Western medical, etc); 1b) Reasoning for treatment provided, literature sources, and/or consensus methods, with references where appropriate; 1c) Extent to which treatment was varied; 2a) Number of needle insertions per subject per session (mean and range where relevant) ; 2b) Names (or location if no standard name) of points used (uni/bilateral) ; 2c) Depth of insertion, based on a specified unit of measurement; 2d) Response sought (e.g. de qi or muscle twitch response); 2e) Needle stimulation (e.g. manual，electrical) ; 2f) Needle retention time; 2g) Needle type (diameter, length, and manufacturer) ; 3a) Number of treatment sessions; 3b) Frequency and duration of treatment sessions; 4a) Details of other interventions administered to the acupuncture group (e.g. moxibustion, cupping, herbs, exercises) ; 4b) Setting and context of treatment, including instructions to practitioners, and information and explanations to patients; 5) Description of participating acupuncturists (qualification or professional affiliation, other relevant experience) ; 6a) Rationale for the control or comparator in the context of the research question, with sources that justify this choice; 6b) Precise description of the control or comparator. If sham acupuncture or any other type of acupuncture-like control is used, provide details as for Items 1 to 3 above. N, not adequately reported; Y, adequately reported.
