# Supplementary material for: Acupuncture for post-stroke depression: a systematic review and network meta-analysis
Source: BMC Psychiatry. 2023 May 4;23:314. doi: 10.1186/s12888-023-04749-1 (PMC10161596; doi:10.1186/s12888-023-04749-1)
Supplement: Supplementary file 3 — Supplementary material 3. The results of ROB and STRICTA assessment for included studies [file 12888_2023_4749_MOESM3_ESM.docx]

**Supplementary Appendix 4.**

**The results of direct and indirect comparison**

|  | **Direct comparison** | **Indirect comparison** | **Network result** | **P value** |
| --- | --- | --- | --- | --- |
| AC with TCM vs. AC | 4.5 (-3.90, 13.00) | 2.8 (-1.50, 7.10) | 3.2 (-0.57, 6.90) | 0.725 |
| AC with TCM vs. WM | 6.4 (2.40, 10.00) | 8.0 (-0.52, 17.00) | 6.7 (3.20, 10.00) | 0.724 |
| TCM vs. AC | 2.7 (-5.80, 11.00) | 1.6 (-5.90, 9.20) | 1.9 (-3.40, 7.30) | 0.848 |
| TCM vs. WM | 4.2 (-3.90, 12.00) | 5.8 (-1.80, 13.00) | 5.4 (0.14, 11.00) | 0.770 |
| AC with WM vs. AC | 4.5 (-0.96, 9.90) | -0.15 (-3.30, 3.00) | 0.85 (-1.90, 3.60) | 0.144 |
| AC vs. WM | 3.8 (2.00, 5.60) | 2.1 (-6.30, 11.00) | 3.5 (1.80, 5.20) | 0.701 |
| WM vs. UC | 8.5 (0.28, 17.00) | 4.8 (1.10, 8.50) | 5.7 (2.30, 9.10) | 0.408 |
