# Supplementary material for: Acupuncture for post-stroke depression: a systematic review and network meta-analysis
Source: BMC Psychiatry. 2023 May 4;23:314. doi: 10.1186/s12888-023-04749-1 (PMC10161596; doi:10.1186/s12888-023-04749-1)
Supplement: Supplementary file 4 — Supplementary material 4. The results of direct and indirect comparison [file 12888_2023_4749_MOESM4_ESM.docx]

**Supplementary Appendix 8. The forest plots for all direct pairwise meta-analysis**

***1. AC vs WM***


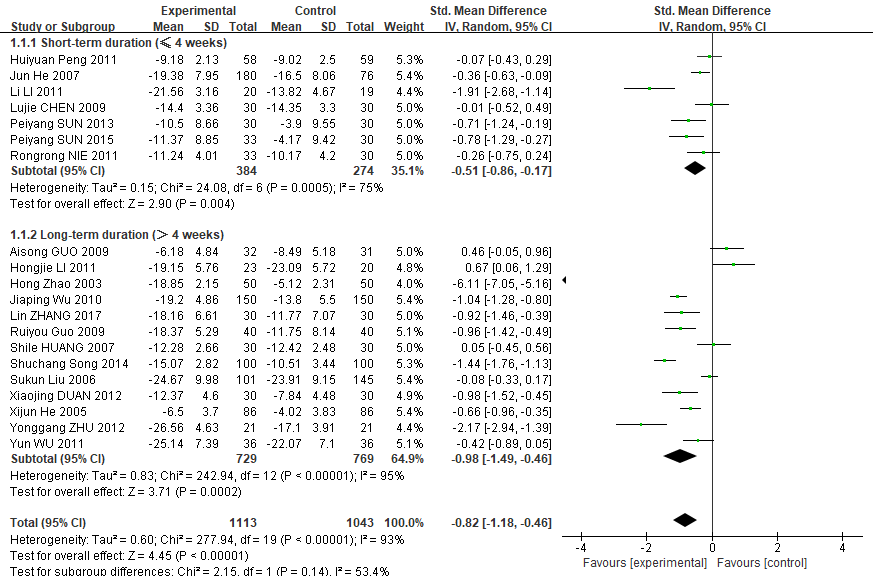


***2. AC with WM vs WM***

***
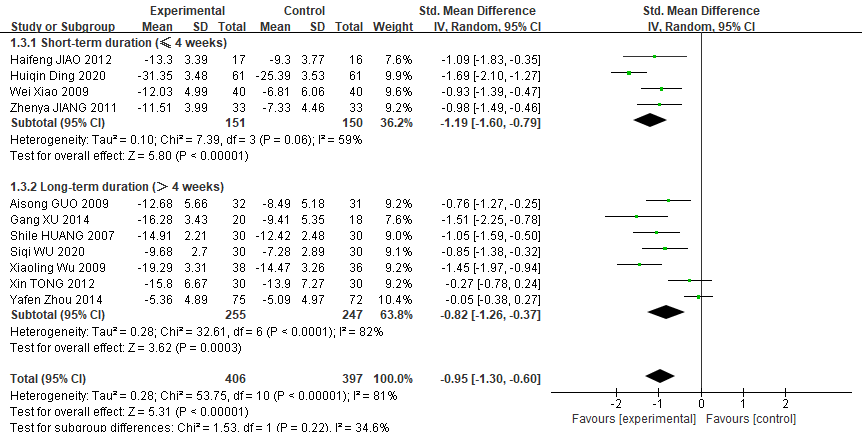
***

***3. AC vs UC***


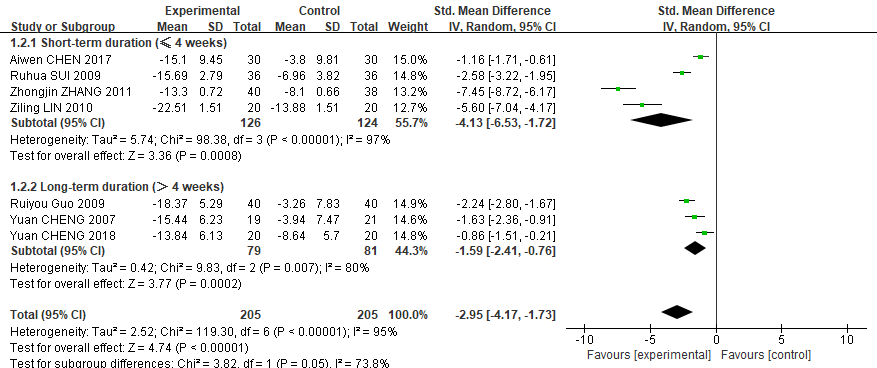


***4. AC with TCM vs WM***

***
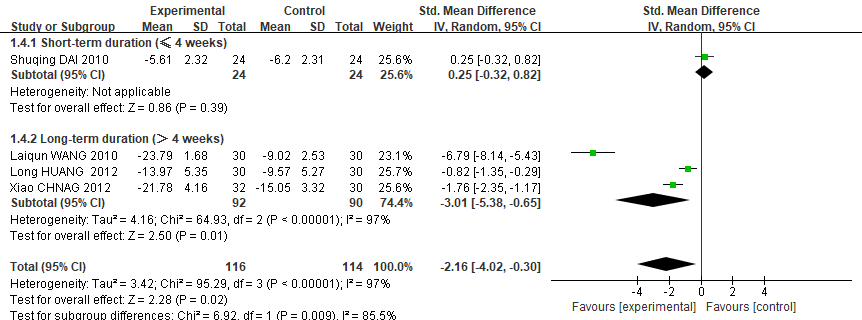
***

***5. AM with WM vs WM***

***
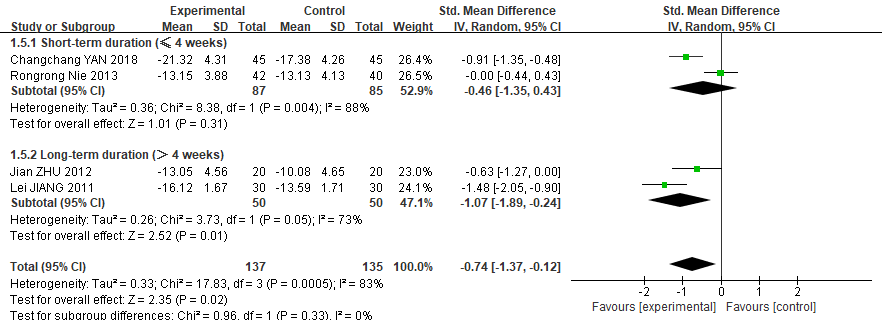
***

***6. AC with WM vs AC***

***
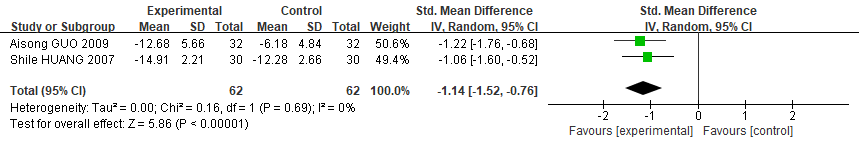
***

***7. AC with TCM vs TCM***

***
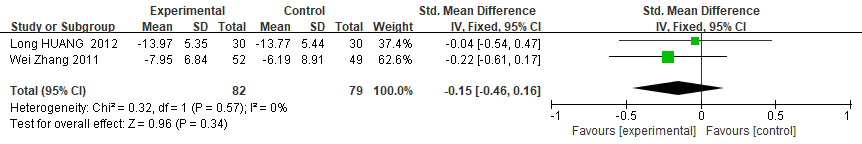
***

***8. AC with TCM with WM vs WM
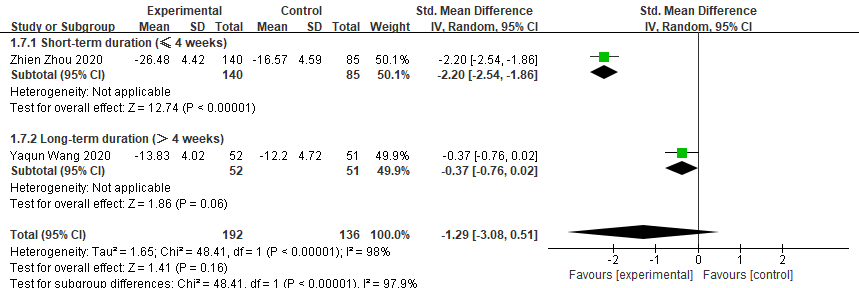
***

***9. AC with RTMS vs AC***

***
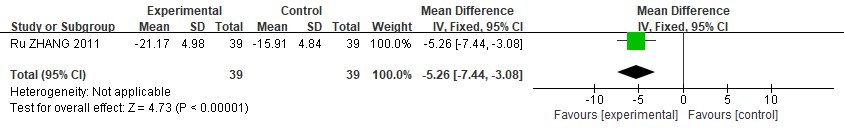
***

***10. AC with TCM vs AC***

***
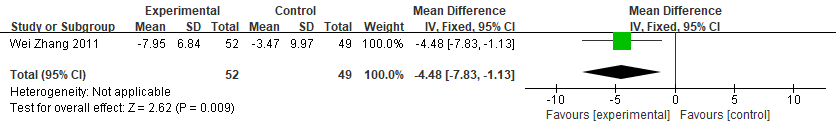
***

***11. TCM vs AC***

***
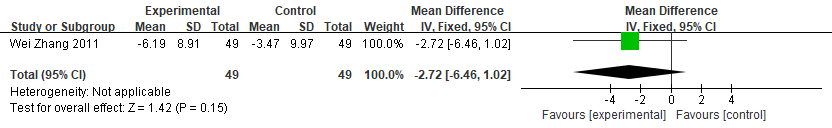
***

***12. AC with CT vs WM***

***
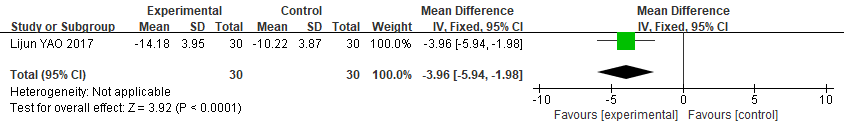
***

***13. AC with TJ vs WM***

***
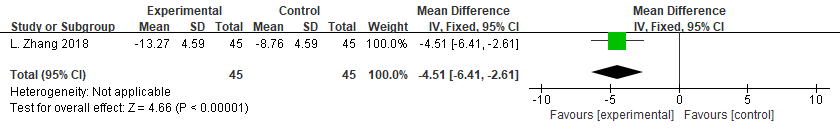
***

***14. AM with WM vs AM***

***
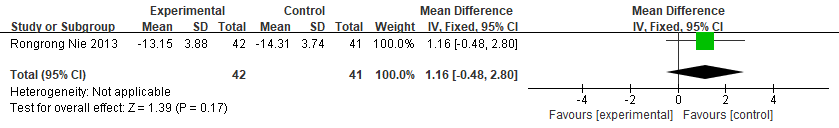
***

***15. AM vs WM***

***
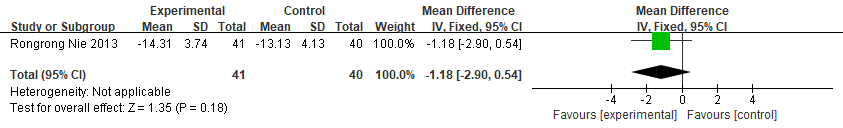
***

***16. TCM vs WM***

***
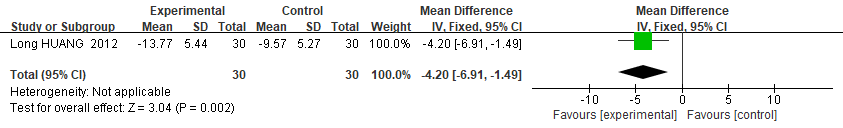
***

***17. WM vs UC***

***
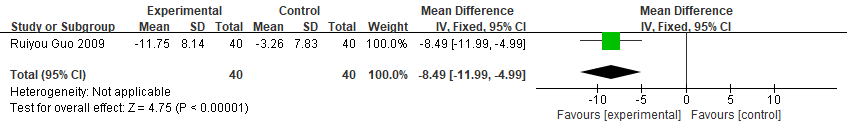
***
