# Supplementary material for: Acupuncture for post-stroke depression: a systematic review and network meta-analysis
Source: BMC Psychiatry. 2023 May 4;23:314. doi: 10.1186/s12888-023-04749-1 (PMC10161596; doi:10.1186/s12888-023-04749-1)
Supplement: Supplementary file 5 — Supplementary material 5. The figure of results of analysis of heterogeneity [file 12888_2023_4749_MOESM5_ESM.docx]

**Supplementary Appendix 9. The main point of acupuncture of included studies.**

| **ID** | **Study** | **The main** **acupoints** |
| --- | --- | --- |
| 5001 | Xin TONG 2012 | Bai-hui, Si-shen-cong, Shui-gou, Yin-tang, Nei-guan, San-yin-jiao, Tai-chong |
| 5076 | Yanxiang LIU 2010 | Ben-shen, Shen-ting, Nei-guan, Gongsun |
| 5245 | Siqi WU 2020 | Shen-ting, Bai-hui, Nei-guan, Waiguan, He-gu, Lao-gong, Tai-chong, Yong-quan, Taixi, Kun-lun, Zu-san-li, Yin-ling-quan |
| 5250 | Xiaoling Wu 2009 | Ren-zhong, Yin-tang, Bai-hui, Sishengcong, Lieque, Houxi, Shenmai, Zhao-hai, Tai-chong |
| 5357 | Lijun YAO 2017 | Shen-ting, Bai-hui, Benshen, Si-shen-cong |
| 5370 | Lei JIANG 2011 | Frontal line, Si-shen-cong, Bai-hui |
| 5391 | Peiyang SUN 2013 | Bai-hui, Feng-fu, Shen-ting, Shui-gou, Dazhui, Shen-dao |
| 5390 | Peiyang SUN 2015 | Bai-hui, Feng-fu, Shen-ting, Shui-gou, Dazhui, Shen-dao |
| 5514 | Xiao CHNAG 2012 | Bai-hui, Tai-chong, Shen-men, Feng-long, San-yin-jiao |
| 5543 | Zhongjin ZHANG 2011 | Bai-hui, Yin-tang, Shen-ting, Nei-guan, Tai-chong, Shen-men, San-yin-jiao, Shui-gou |
| 5597 | Guibo ZHANG 2010 | Shui-gou, Bai-hui, Yin-tang, Si-shen-cong, Shen-men, He-gu, Nei-guan, San-yin-jiao, Tai-chong |
| 5624 | Ru ZHANG 2011 | Feng-chi, Binao, Quchi, Waiguan, Siqiang, Zu-san-li, Yongquan |
| 5668 | Lin ZHANG 2017 | Bai-hui, Si-shen-cong, Shen-ting, Yin-tang, Shen-men, Nei-guan, Tai-chong, He-gu, Zu-san-li, San-yin-jiao, Fenglong, Shen-men, Pizhixia |
| 5700 | Gang XU 2014 | Wangu, Bai-hui, Fengchi, Tian-zhu, Nei-guan, Shen-men, Si-shen-cong, Zu-san-li, San-yin-jiao |
| 5738 | Shuqing DAI 2010 | Si-shen-cong, Yin-tang, Nei-guan, Shen-men |
| 5794 | Jian ZHU 2012 | Frontal line, Si-shen-cong, Bai-hui |
| 5805 | Yonggang ZHU 2012 | Shen-men, Qiunao, Pizhixia |
| 5995 | Li LI 2011 | Bai-hui, Yin-tang, Si-shen-cong, Nei-guan, Shen-men |
| 6012 | Hongjie LI 2011 | Bai-hui, Yin-tang, Si-shen-cong, Tai-chong, Shen-men, Nei-guan, San-yin-jiao, Taixi, Xinyu |
| 6069 | Ziling LIN 2010 | Bai-hui, Shen-ting, Nei-guan, Shen-men, Qi-hai, San-yin-jiao, Tai-chong |
| 6242 | Yun WU 2011 | Bai-hui, Si-shen-cong, Nei-guan, Shui-gou, San-yin-jiao, Jiquan, Fengchi, Quchi, He-gu, Chize, Zu-san-li, Tai-chong, Gongsun, Zhao-hai, Huantiao |
| 6247 | Haifeng JIAO 2012 | Bai-hui |
| 6372 | Laiqun WANG 2010 | Zu-san-li, San-yin-jiao, Nei-guan, Bai-hui, Shui-gou, Si-shen-cong, Yin-tang, Tai-chong, Shen-men |
| 6304 | Changchang YAN 2018 | Frontal line, Bai-hui, Si-shen-cong |
| 6545 | Yuan CHENG 2007 | Zhong-wan, Xiawan, Qi-hai, Huaroumen, Wai-ling, Daheng |
| 6546 | Yuan CHENG 2018 | Zhong-wan, Xiawan, Qi-hai, Guan-yuan, Hua-rou-men, Wailing, Daheng |
| 6588 | Rongrong NIE 2011 | Zhong-wan, Zhang-men, Zu-san-li, Yin-ling-quan |
| 6662 | Zhiwei SU 2010 | Bai-hui, Qian-ding, Xin-hui, Shang-xing, Shen-ting |
| 6703 | Guomin JIANG 2010 | Bai-hui, Feng-fu, Feng-chi, Yin-tang |
| 6705 | Zhenya JIANG 2011 | Bai-hui, Yin-tang |
| 6839 | Xiaobing ZHAO 2012 | Bai-hui, Ren-zhong, Shen-ting, Si-shen-cong, Guan-yuan, Qi-hai, Zu-san-li, Xiayinjiao |
| 6942 | Aisong GUO 2009 | Bai-hui, Shen-ting, Yin-tang, Si-shen-dong, He-gu, Tai-chong |
| 7043 | Aiwen CHEN 2017 | Bai-hui, Si-shen-cong, Nei-guan, He-gu, Tai-xi, Tai-chong, Zu-san-li, Xue-hai |
| 7083 | Lujie CHEN 2009 | Ren-zhong, Nei-guan, Tai-chong, Tong-li, Da-ling, He-gu, Quchi, Yang-ling-quan, Xuan-zhong, Zu-san-li, Qi-hai, Xue-hai |
| 7089 | Ruhua SUI 2009 | Shen-tang, Geguan, Hunmen, Yanggang, Shen-men, Huan-taio, Tai-chong |
| 7182 | Wa GAO 2017 | Yin-tang, Shen-men, Qiu-xu, Tai-chong |
| 7213 | Shile HUANG 2007 | Bai-hui, Qiang-jian, Ben-shen, Tian-chong |
| 7271 | Long HUANG 2012 | Bai-hui, Yin-tang, Si-sheng-cong, Feng-fu, Nei-guan, San-yin-jiao, He-gu, Zu-san-li, Tai-chong |
| 5604 | Aibing ZHANG 2009 | Bai-hui, Tai-yang, Feng-chi, San-yin-jiao, Nei-guan, Zu-san-li, Qi-hai, Xue-hai |
| 6169 | Xiaojing DUAN 2012 | Shang-xing, Tan-zhong, Zhong-wan, Tai-chong |
| 6522 | Fengkui ZHU 2010 | Ben-shen, Shen-ting, Bai-hui, Tai-bai, Tai-chong |
| 6279 | Yahui WANG 2016 | Zhong-wan, Xiawan, Qi-hai, Guan-yuan, Tian-shu, Da-heng, Hua-rou-men, Wai-ling, Shang-Feng-shi-dian, Shang-Feng-shi-wai-dian, Xia-Feng-shi-dian, Xia-Feng-shi-xia-dian |
| 5400 | Wenge SUN 2012 | Wu-yu, Jian-jing, Jian-liao, Jian-zhen |
| 1095 | Ruiyou Guo 2009 | Gong-sun, Nei-guan, Zhu-lin-qi, Wai-guan, Hou-xi, Shen-mai, Lie-que, Zhao-hai |
| 5113 | Sukun Liu 2006 | Si-shen-cong, Anmian, Nei-guan, Shen-men, Zu-san-li, San-yin-jiao, Tai-chong, Zhao-hai, Shen-mai |
| 5273 | Yafen Zhou 2014 | He-gu, Tai-chong, Bai-hui, Yin-tang, Shen-ting, Si-shen-cong |
| 5690 | Huiyuan Peng 2011 | Nie-San-Zhen, Nei-guan, Zu-san-li, Feng-long, Tai-chong |
| 5677 | Wei Zhang 2011 | Si-shen-cong, Quchi, Waiguan, Zu-san-li, Yang-ling-quan, Kun-lun, Tai-chong |
| 6270 | Yaqun Wang 2020 | Bai-hui, Fengfu, Shen-ting, Da-zhui, Shui-gou, Shen-dao |
| 2649 | Rongrong Nie 2013 | He-gu, Tai-chong, Bai-hui, Yin-tang |
| 6862 | Hong Zhao 2003 | Nei-guan, Ren-zhong, San-yin-jiao, Fengchi, Wan-gu, Tianzhu, Ji-quan, Chi-ze, Wei-zhong, He-gu, Bai-hui |
| 6615 | Wei Xiao 2009 | Feng-fu, Ya-men, Xian-ao-hu |
| 4926 | Huiqin Ding 2020 | Nei-guan, Shen-men, He-gu, San-yin-jiao, Si-shen-cong |
| 5242 | Jiaping Wu 2010 | Bai-hui, Ben-shen, Shen-ting, Si-shen-cong, Shen-men |
| 3169 | Shuchang Song 2014 | Bai-hui, Si-shen-cong, Shen-ting, Shi-er-jing |
| 4457 | L. Zhang 2018 | Bai-hui, Si-shen-cong, Shen-ting, Yin-tang, Shen-men, Nei-guan, Tai-chong, He-gu, Chi-ze, Qu-chi, Shou-san-li, Feng-shi, Xue-hai, Yang-ling-quan, Qiu-xu |
| 5298 | Zhien Zhou 2020 | Bai-hui |
| 1175 | Jun He 2007 | Nei-guan, Shui-gou, Bai-hui, Yin-tang, San-yin-jiao |
| 1193 | Xijun He 2005 | Ren-zhong, Nei-guan, Tai-chong, Shen-men |
