# Supplementary figures and images for: Acupuncture for post-stroke depression: a systematic review and network meta-analysis
Source: BMC Psychiatry. 2023 May 4;23:314. doi: 10.1186/s12888-023-04749-1 (PMC10161596; doi:10.1186/s12888-023-04749-1)

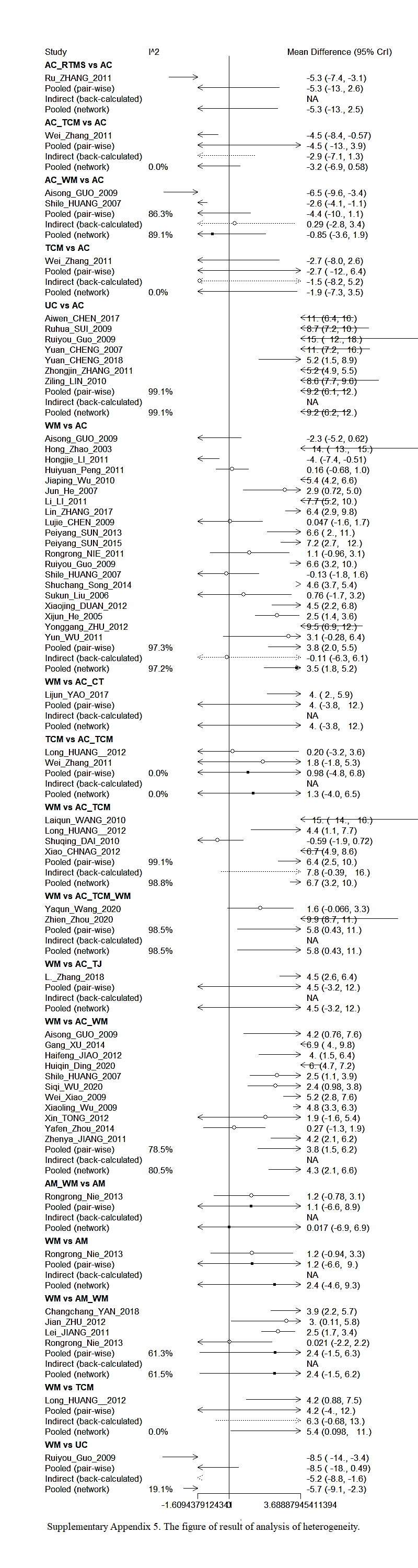

Supplement: Supplementary file 6 — Supplementary material 6. The figure of node-splitting analysis of inconsistency [file 12888_2023_4749_MOESM6_ESM.png]

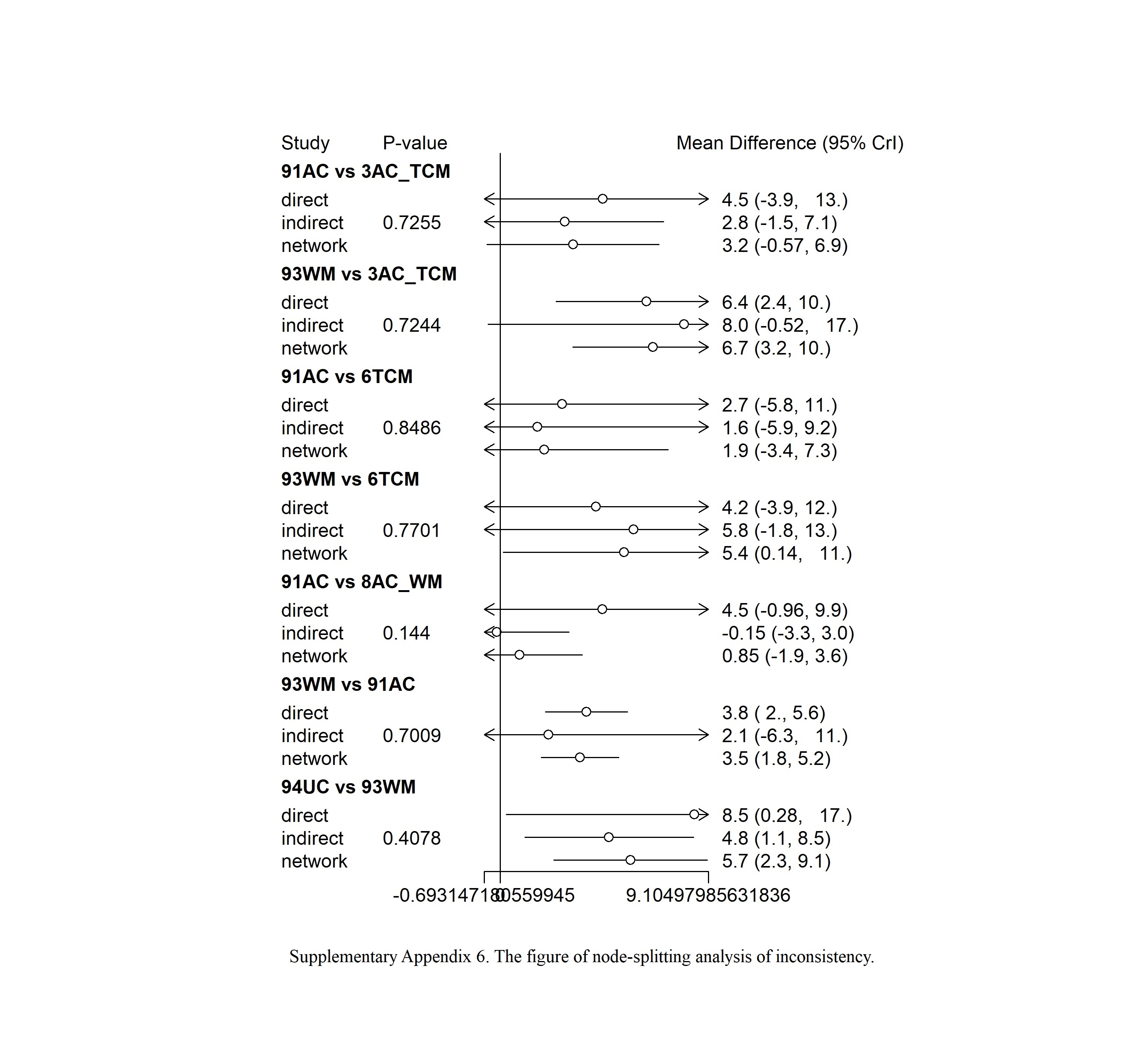

Supplement: Supplementary file 7 — Supplementary material 7. The figure of SUCRA [file 12888_2023_4749_MOESM7_ESM.png]

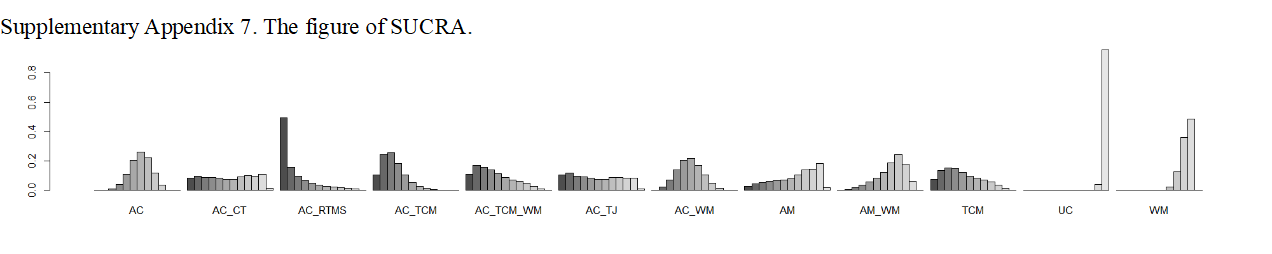

Supplement: Supplementary file 8 — Supplementary material 8. The forest plots for all direct pairwise meta-analysis [file 12888_2023_4749_MOESM8_ESM.png]
