# Supplementary material for: Acupuncture for post-stroke depression: a systematic review and network meta-analysis
Source: BMC Psychiatry. 2023 May 4;23:314. doi: 10.1186/s12888-023-04749-1 (PMC10161596; doi:10.1186/s12888-023-04749-1)
Supplement: Supplementary file 9 — Supplementary material 9. The main point of acupuncture of included studies [file 12888_2023_4749_MOESM9_ESM.docx]

**Supplemental Appendix 2. Search strategy**

***Cochrane Library***

#1 [mh ^"cerebrovascular disorders"] or [mh "basal ganglia cerebrovascular disease"] or [mh "brain ischemia"] or [mh "carotid artery diseases"] or[mh "cerebral small vessel diseases"] or[mh "intracranial arterial diseases"] or[mh "intracranial embolism and thrombosis"] or[mh "intracranial hemorrhages"] or[mh ^stroke] or[mh "brain infarction"] or[mh ^"stroke, lacunar"] or[mh ^"vasospasm, intracranial"] or [mh ^"vertebral artery dissection"]

#2 (stroke or poststroke or "post-stroke" or cerebrovasc* or brain next vasc* or cerebral next vasc* or cva* or apoplex* or SAH):ti,ab

#3 ((brain* or cerebr* or cerebell* or intracran* or intracerebral) near/5 (isch*emi* or infarct* or thrombo* or emboli* or occlus*)):ti,ab

#4 ((brain* or cerebr* or cerebell* or intracerebral or intracranial or subarachnoid) near/5 (haemorrhage* or hemorrhage* or haematoma* or hematoma* or bleed*)):ti,ab

#5 [mh ^hemiplegia] or [mh ^paresis]

#6 (hemipleg* or hemipar* or paresis or paretic):ti,ab

#7 [mh ^"brain injuries"] or [mh ^"brain injury, chronic"]

#8 #1 or #2 or #3 or #4 or #5 or #6 or #7

#9 [mh ^acupuncture] or [mh ^"acupuncture therapy"] or [mh ^"acupuncture analgesia"] or [mh ^"acupuncture, ear"] or [mh ^auricular acupuncture] or [mh ^head acupuncture] or [mh ^fire acupuncture] or [mh ^manual acupuncture] or [mh ^electroacupuncture] or [mh ^meridians] or [mh ^"acupuncture points"] or [mh ^"trigger points"]

#10 (acupuncture* or electroacupuncture or "electro-acupuncture" or acupoint* or meridians or needling):ti,ab

#11 ((meridian or non-meridian or trigger) near/10 point*):ti,ab

#12 #9 or #10 or #11

#13 #8 and #12

***PubMed (Medline)***

1. cerebrovascular disorders/ or exp basal ganglia cerebrovascular disease/ or exp brain ischemia/ or exp carotid artery diseases/ or exp

cerebral small vessel diseases/ or exp intracranial arterial diseases/ or exp "intracranial embolism and thrombosis"/ or exp intracranial

hemorrhages/ or stroke/ or exp brain infarction/ or stroke, lacunar/ or vasospasm, intracranial/ or vertebral artery dissection/

2. (stroke or poststroke or post-stroke or cerebrovasc$ or brain vasc$ or cerebral vasc$ or cva$ or apoplex$ or SAH).tw.

3. ((brain$ or cerebr$ or cerebell$ or intracran$ or intracerebral) adj5 (isch?emi$ or infarct$ or thrombo$ or emboli$ or occlus$)).tw.

4. ((brain$ or cerebr$ or cerebell$ or intracerebral or intracranial or subarachnoid) adj5 (haemorrhage$ or hemorrhage$ or haematoma$

or hematoma$ or bleed$)).tw.

5. hemiplegia/ or exp paresis/

6. (hemipleg$ or hemipar$ or paresis or paretic).tw.

7. brain injuries/ or brain injury, chronic/

8. or/1-7

9. acupuncture/ or acupuncture therapy/ or acupuncture analgesia/ or acupuncture, ear/ or auricular acupuncture/ or head acupuncture/ or fire acupuncture/ or manual acupuncture/ or electroacupuncture/ or meridians/ or acupuncture points/ or trigger points/

10. (acupuncture$ or electroacupuncture or electro-acupuncture or acupoint$ or meridians or needling).tw.

11. ((meridian or non-meridian or trigger) adj10 point$).tw.

12. 9 or 10 or 11

13. 8 and 12

14. randomized controlled trials as Topic/

15. random allocation/

16. controlled clinical trials as Topic/

17. control groups/

18. clinical trials as topic/ or clinical trials, phase i as topic/ or clinical trials, phase ii as topic/ or clinical trials, phase iii as topic/ or clinical

trials, phase iv as topic/

19. double-blind method/

20. single-blind method/

21. placebos/

22. placebo eGect/

23. cross-over studies/

24. randomized controlled trial.pt.

25. controlled clinical trial.pt.

26. (clinical trial or clinical trial phase i or clinical trial phase ii or clinical trial phase iii or clinical trial phase iv).pt.

27. (random$ or RCT or RCTs).tw.

28. (controlled adj5 (trial$ or stud$)).tw.

29. (clinical$ adj5 trial$).tw.

30. ((control or treatment or experiment$ or intervention) adj5 (group$ or subject$ or patient$)).tw.

31. (quasi-random$ or quasi random$ or pseudo-random$ or pseudo random$).tw.

32. ((control or experiment$ or conservative) adj5 (treatment or therapy or procedure or manage$)).tw.

33. ((singl$ or doubl$ or tripl$ or trebl$) adj5 (blind$ or mask$)).tw.

34. (cross-over or cross over or crossover).tw.

35. (placebo$ or sham).tw.

36. trial.ti.

37. (assign$ or allocat$).tw.

38. controls.tw.

39. or/14-38

40. 13 and 39

41. exp animals/ not humans/

42. 40 not 41

***EMBASE***

1. cerebrovasculardisease/ or expbasal ganglion hemorrhage/ or expbrain hematoma/ or expbrain hemorrhage/ or expbrain infarction/ or exp brain ischemia/ or exp carotid artery disease/ or cerebral artery disease/ or exp cerebrovascular accident/ or exp intracranial aneurysm/ or exp occlusive cerebrovascular disease/ or stroke unit/ or stroke patient/

2. (stroke or poststroke or post-stroke or cerebrovasc$ or brain vasc$ or cerebral vasc$ or cva$ or apoplex$ or SAH).tw.

3. ((brain$ or cerebr$ or cerebell$ or intracran$ or intracerebral) adj5 (isch?emi$ or infarct$ or thrombo$ or emboli$ or occlus$)).tw.

4. ((brain$ or cerebr$ or cerebell$ or intracerebral or intracranial or subarachnoid) adj5 (haemorrhage$ or hemorrhage$ or haematoma$ or hematoma$ or bleed$)).tw.

5. hemiparesis/ or hemiplegia/ or paresis/

6. (hemipleg$ or hemipar$ or paresis or paretic).tw.

7. brain injury/ or acquired brain injury/

8. or/1-7

9. acupuncture/ or acupuncture therapy/ or acupuncture analgesia/ or acupuncture, ear/ or auricular acupuncture/ or head acupuncture/ or fire acupuncture/ or manual acupuncture/ or electroacupuncture/ or meridians/ or acupuncture points/ or trigger points/

10. (acupuncture$ or electroacupuncture or electro-acupuncture or acupoint$ or meridians or needling).tw.

11. ((meridian or non-meridian or trigger) adj10 point$).tw.

12. 9 or 10 or 11

13. 8 and 12

14. randomized controlled trial/ or "randomized controlled trial (topic)"/

15. randomization/

16. controlled clinical trial/ or "controlled clinical trial (topic)"/

17. control group/ or controlled study/

18. clinical trial/ or "clinical trial (topic)"/ or phase 1 clinical trial/ or phase 2 clinical trial/ or phase 3 clinical trial/ or phase 4 clinical trial/

19. crossover procedure/

20. double blind procedure/

21. single blind Procedure/ or triple blind procedure/

22. placebo/ or placebo eGect/

23. (random$ or RCT or RCTs).tw.

24. (controlled adj5 (trial$ or stud$)).tw.

25. (clinical$ adj5 trial$).tw.

26. ((control or treatment or experiment$ or intervention) adj5 (group$ or subject$ or patient$)).tw.

27. (quasi-random$ or quasi random$ or pseudo-random$ or pseudo random$).tw.

28. ((control or experiment$ or conservative) adj5 (treatment or therapy or procedure or manage$)).tw.

29. ((singl$ or doubl$ or tripl$ or trebl$) adj5 (blind$ or mask$)).tw.

30. (cross-over or cross over or crossover).tw.

31. (placebo$ or sham).tw.

32. trial.ti.

33. (assign$ or allocat$).tw.

34. controls.tw.

35. or/14-34

36. 13 and 35

37.(exp animals/ or exp invertebrate/ or animal experiment/ or animal model/ or animaltissue/ or animal cell/ or nonhuman/) not(human/

or normal human/ or human cell/)

38. 36 not 37

***China National Knowledge Infrastructure (CNKI)(Chinese database)***

SU=(' Zhong Feng '+' Cu Zhong '+' Nao Xue Guan '+' Nao Geng Se '+' Nao Geng Si '+' Nao Xue Shuan '+' Nao Shuan Sai '+' Nao Chu Xie '+' Nao Yi Xue '+' Zhu Wang Mo Xia Qiang Chu Xie ') AND (' Zhen Jiu '+' Zhen Ci '+' Dian Zhen '+' Mang Zhen '+' Pi Xia Zhen '+' Huo Zhen '+' Tou Zhen '+' Er Zhen '+' Shou Nian Zhen '+' Zhen Dao '+' Chang Zhen '+' Ju Zhen '+' Tou Pi Zhen '+' Ti Zhen '+' Wen Zhen '+' Tou Ci '+' Ju Ci '+' Zhen Fa '+' Ci Fa '+' Yan Zhen '+' Ci Ji Zhen '+' Hao Zhen '+' Miu Ci '+' Pi Nei Zhen '+' Chan Zhen '+' Yuan Zhen '+' Chi Zhen '+' Feng Zhen '+' Pi Zhen '+' Yuan Li Zhen '+' Da Zhen ')

***Wanfang Database (Chinese database)***

(title or key work: " Zhong Feng "+" Cu Zhong "+" Nao Xue Guan "+" Nao Geng Se "+" Nao Geng Si "+" Nao Xue Shuan "+" Nao Shuan Sai "+" Nao Chu Xie "+" Nao Yi Xue "+" Zhu Wang Mo Xia Qiang Chu Xie ") AND (title or key work: " Zhen Jiu "+" Zhen Ci "+" Dian Zhen "+" Mang Zhen "+" Pi Xia Zhen "+" Huo Zhen "+" Tou Zhen "+" Er Zhen "+" Shou Nian Zhen "+" Zhen Dao "+" Chang Zhen "+" Ju Zhen "+" Tou Pi Zhen "+" Ti Zhen "+" Wen Zhen "+" Tou Ci '"+" Ju Ci "+" Zhen Fa "+" Ci Fa "+" Yan Zhen "+" Ci Ji Zhen "+" Hao Zhen "+" Miu Ci "+" Pi Nei Zhen "+" Chan Zhen "+" Yuan Zhen "+" Chi Zhen "+" Feng Zhen "+" Pi Zhen "+" Yuan Li Zhen "+" Da Zhen ")

***Chongqing VIP Database (CQVIP) (Chinese database)***

(M=( “Zhong Feng” OR “Cu Zhong OR Nao Xue Guan” OR “Nao Geng Se” OR “Nao Geng Si” OR “Nao Xue Shuan” OR “Nao Shuan Sai” OR “Nao Chu Xie” OR “Nao Yi Xue” OR “Zhu Wang Mo Xia Qiang Chu Xie”) AND M=( “Zhen Jiu” OR “Zhen Ci” OR “Dian Zhen” OR “Mang Zhen” OR “Pi Xia Zhen” OR “Huo Zhen” OR “Tou Zhen” OR “Er Zhen” OR “Shou Nian Zhen” OR “Zhen Dao” OR “Chang Zhen” OR “Ju Zhen” OR “Tou Pi Zhen” OR “Ti Zhen” OR “Wen Zhen” OR “Tou Ci” OR “Ju Ci” OR “Zhen Fa” OR “Ci Fa” OR “Yan Zhen” OR “Ci Ji Zhen” OR “Hao Zhen” OR “Miu Ci” OR “Pi Nei Zhen” OR “Chan Zhen” OR “Yuan Zhen” OR “Chi Zhen” OR “Feng Zhen” OR “Pi Zhen” OR “Yuan Li Zhen” OR “Da Zhen”))
